# Prickle4 Drives Microenvironmental Remodeling and Resistance to Parp Inhibition in IDH‐Mutant Glioma

**DOI:** 10.1002/advs.202503866

**Published:** 2025-11-14

**Authors:** Ju Yang, Hua Yang, Yifan Yuan, Chenyang Zhang, Ziwei Fu, Yanyan Chen, Yinghong Xiong, Shuyu Chen, Kexin Ling, Ying Liu, Jason T. Huse, Bo Chen, Timothy A. Chan, Zengxin Qi, Zhao Zhang, Xiuping Liu, Yuxiang Wang

**Affiliations:** ^1^ Department of Pathology School of Basic Medical Sciences Shanghai Fifth People's Hospital Fudan University Shanghai 200040 China; ^2^ Department of Pathology School of Basic Medical Sciences Fudan University Shanghai 200040 China; ^3^ Department of Neurosurgery National Center for Neurological Disorders Huashan Hospital Fudan University Shanghai 200040 China; ^4^ Departments of Pathology and Translational Molecular Pathology University of Texas MD Anderson Cancer Center Houston TX 77030 USA; ^5^ Keymed Biosciences (Chengdu) Limited Chengdu Sichuan 610219 China; ^6^ Center for Immunotherapy and Precision Immuno‐Oncology Cleveland Clinic Cleveland Ohio 44195 USA; ^7^ Department of Neurosurgery Huashan Hospital Shanghai Medical College Fudan University Shanghai 200040 China; ^8^ National Center for Neurological Disorders Shanghai 200040 China; ^9^ Shanghai Key Laboratory of Brain Function and Restoration and Neural Regeneration Shanghai 200040 China; ^10^ MOE Key Laboratory of Metabolism and Molecular Medicine Department of Biochemistry and Molecular Biology School of Basic Medical Sciences Fudan University Shanghai 200040 China; ^11^ Department of Pathology School of Basic Medical Sciences Fudan University & Shanghai Pudong Hospital Fudan University Pudong Medical Center Shanghai 200032 China

**Keywords:** angiogenesis, glioma, IDH, PARP

## Abstract

Mutations in isocitrate dehydrogenase (IDH) genes sensitize gliomas to PARP inhibition (PARPi) by inducing epigenetic reprogramming of DNA damage repair circuits. However, tumors treated with PARPi eventually relapse despite initial responsiveness. In this study, it is demonstrated that the anti‐angiogenic agent lenvatinib synergizes effectively with PARPi, resulting in substantial tumor regression and significantly extended survival. Genomic analysis of tumors reveals that PARPi induces widespread transcriptomic changes that are predominantly pro‐inflammatory, thereby promoting tumor angiogenesis. Prickle4, a planar cell polarity protein, is identified as a critical mediator of PARPi‐induced neovascularization. Targeting Prickle4 effectively overcomes PARPi resistance in these tumors. Collectively, these findings identified the Prickle4‐mediated microenvironmental remodeling as the key resistance mechanism to PARPi, and support the therapeutic promise of multimodal therapy combining PARPi with anti‐angiogenic agents for glioma treatment.

## Graphical abstract

IDH‐mutant gliomas exhibit sensitivity to PARP inhibitors (PARPi) owing to oncometabolite 2‐HG‐induced DNA repair defects. PARPi treatment induces resistance by upregulating Prickle4, which promotes VEGF‐driven angiogenesis. Combining PARPi veliparib with anti‐angiogenic lenvatinib blocks this pathway, causing tumor regression and prolonged survival in models, revealing a new adaptive resistance mechanism and supporting a promising combination strategy.

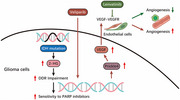

## 1 Introduction

DNA damage arising during cell propagation or due to exogenous sources of agents, such as ionizing radiation, is managed by intrinsic repair mechanisms. These machineries identify the class of DNA damage and initiate the corresponding repair pathways.^[^ ^1^ ^]^ While these pathways have evolved to be precise and specific, their interaction and cooperation are vital for cell survival.^[^ ^2^, ^3^ ^]^ In cancer, the increased burden of DNA damage results from less effective fidelity control during replication and higher levels of oxidation due to oncogenic mutations, necessitating heightened DNA repair activities for cancer cells to manage stress. Understanding how these repair pathways interact is crucial for cancer treatment. For instance, the class of poly ADP‐ribose polymerase (PARP) proteins actively perform PARylation and release nicotinamide, playing roles in base excision repair (BER) and single‐strand break (SSB) repair. With the loss of repairing activity of PARP, the cell is in of struggle for survival, alternatively promoting double‐strand breaks (DSB) and subsequent BRCA‐dependent repair by homologous recombination (HR). This functional cooperation underlies the strategy of targeting BRCA‐deficient cancer cells with PARP inhibitors, while BRCA‐proficient cells survive within an appropriate therapeutic window.^[^ ^4^ ^]^ The concept of “BRCAness”, illuminated by the BRCA‐PARP synthetic lethal approach, describes how other tumors share traits with BRCA1/2 mutant carriers, leading to molecular abnormalities in DNA damage response pathways and sensitivity to PARP inhibitors.^[^ ^5^ ^]^ Laboratory investigations, coupled with established bioinformatic methods to assess BRCAness, have provided proof‐of‐concept that BRCAness‐high tumors are more likely to respond to PARP inhibition, as observed in the case of ovarian cancer, breast cancer, and prostate cancer.^[^ ^6^ ^]^

Gliomas are the most common primary brain tumors, typically managed with surgical resection followed by chemo‐ and/or radiation therapies as part of the standard of care.^[^ ^7^ ^]^ Investigational targeted therapies have shown promise in some cases, yet few have been validated through clinical trials.^[^ ^8^ ^]^ Mutations in isocitrate dehydrogenase (IDH) genes define the subclass of gliomas that display overall lower malignancy and better prognosis. However, the infiltrative nature of these tumors often leads to nearly inevitable local recurrence featuring unmanageable growth. The neomorphic mutant IDH (referred to as “IDHmut”) produces 2‐hydroxyglutarate (2‐HG), which competitively inhibits α‐ketoglutarate (α‐KG)‐dependent dioxygenases, including histone and DNA demethylases, thereby inducing global epigenomic changes.^[^ ^9^, ^10^ ^]^ While the efficacy of IDHmut‐specific inhibitors has been evaluated in glioma models, their clinical translation has been accompanied by controversial evidence.^[^ ^11^, ^12^, ^13^, ^14^ ^]^ Nevertheless, clinical studies have shown promising results, albeit requiring further clarification on optimal timing, dosage, and other parameters.^[^ ^15^, ^16^ ^]^ Mixed results yielded from strategies targeting IDHmut have prompted the exploration of alternative approaches proposed and primarily tested in laboratory and/or preclinical settings.^[^ ^17^ ^]^ Our previous study showed that IDHmut expression led to increased genomic instability.^[^ ^11^ ^]^ Further investigations suggest that this instability may stem from defective DNA damage repair (DDR) via the homologous recombination (HR) pathway, aligning with the PARP synthetic lethality model.^[^ ^14^, ^18^, ^19^ ^]^ In multiple animal models of IDHmut brain tumors, PARP inhibition effectively inhibits tumor growth, with enhanced efficacy observed when combined with ionizing radiation.^[^ ^18^ ^]^ Radiation therapy, being a part of standard treatments for gliomas, however, lacks specificity and poses unwanted damage to the nontumorous brain tissues. Therefore, a synergistic agent with PARP inhibition that offers specificity is of compelling need.

The feasibility of combined PARP and angiogenesis inhibition in IDHmut gliomas remains to be explored. PARP inhibition has been shown to induce alterations in DDR pathways linked to hypoxia and angiogenesis. For instance, hypoxia induces RAD51 destabilization and impairs HR‐mediated DDR efficiency.^[^ ^20^ ^]^ Also, DDR‐inducing agents promote nitric oxide synthase activity and vascular endothelial growth factor (VEGF) expression.^[^ ^21^ ^]^ Indeed, epidemiological studies indicated that patients with chronic DNA damage or deficiency in DDR exhibit a higher rate of tumor angiogenesis.^[^ ^22^, ^23^ ^]^ In a clinical setting, the combination of bevacizumab, an angiogenesis inhibitor, with the PARP inhibitor olaparib has shown promising synergy in a phase III clinical trial for advanced ovarian cancer.^[^ ^24^ ^]^ However, the fundamental mechanisms underlying these interactions remain to be fully elucidated.

In this study, we hypothesized that concurrent inhibition of angiogenesis and PARP has synergistic effects in IDHmut settings. Here, we report that veliparib and lenvatinib significantly inhibited the growth of IDHmut brain tumors and prolonged the survival of IDHmut tumor‐bearing mice. Moreover, PARP inhibition results in alterations of the planar cell polarity pathway, mediated by Prickle4, which constitutes the basis of PARPi resistance. Suppression of Prickle4 effectively overcomes PARPi resistance. Overall, our findings provided evidence supporting the use of veliparib and lenvatinib combination therapy to treat IDHmut gliomas.

## 2 Results

### 2.1 IDH Mutation Status Defines Distinct Microenvironment in IDH Mutant Gliomas

**Figure 1:**
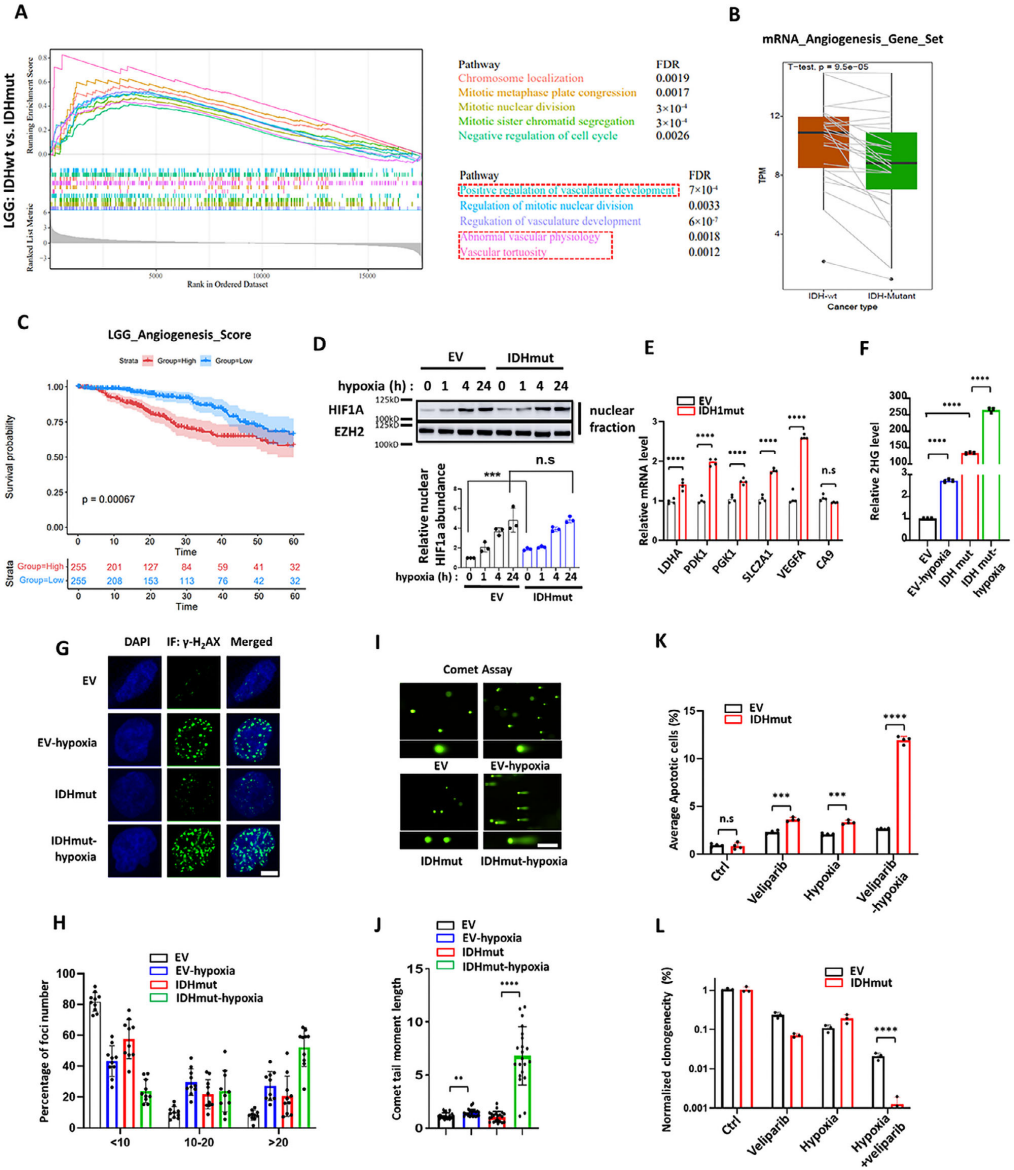
Mutant IDH1 and hypoxia induce DNA damage synergistically in IHA. A) GSEA analyses of significantly enriched gene sets related to angiogenesis. Blue dotted box: pathways related to mitotic activities. Red dotted box: pathways related to vasculature regulation. B) Comparison of average expression of 26 genes in IDHwt and IDHmut tumors. C) Kaplan‐Meier analysis comparing overall survival in angiogenesis score‐high and ‐low patients. *P* values were determined by the log‐rank (Mantel‐Cox) test. D) Western blot analysis of HIF‐1α and EZH2 (loading control) under hypoxia (1% O_2_). Experiments were repeated three times, and the average signal intensity was plotted on bar graphs as mean ± SEM. N = 3. E) mRNA transcript abundance of LDHA, PDK1, PGK1, SLC2A1, VEGFA, and CA9 (internal control) was determined by Real‐time PCR and plotted on bar graphs as mean ± SEM. N = 4. F) Relative intracellular 2‐HG level in IHA‐EV and IHA‐IDH1mut under hypoxic and non‐hypoxic conditions measured by LC‐MS. N = 3. G) Immunofluorescence staining of γ‐H_2_AX (green) in IHA‐EV and IHA‐IDH1mut under hypoxic and non‐hypoxic conditions. Blue: DAPI. Scale bar represents 10 µm. H). Ten independent fields were quantified, and the percentage of average γ‐H_2_AX foci in the nucleus was plotted. N = 10. I) Neutral Comet assays determining DNA breaks in IHA‐EV and IHA‐IDH1mut under hypoxic and non‐hypoxic conditions. Scale bar represents 200 µm. J). The length of comet tails was quantified and represented on the plot. N = 20. K) propidium iodide (PI) positivity of IHA‐EV and IHA‐IDH1mut, treated with veliparib (20 µM) and hypoxia (1% O_2_) was determined by flow cytometry, quantified, and plotted. N = 4. L) Clonogenic survival assays of IHA‐EV and IHA‐IDH1mut cells under the indicated conditions. The colonies were quantified and represented on the bar graph showing the synergistic effect of veliparib and hypoxia, specifically in IHA‐IDH1mut cells. N = 3. ^*^ *p* < 0.05, ^**^ *p* < 0.01, ^***^ *p* < 0.001, ^****^ *p* < 0.0001, ns: not significant.

The impact of IDH mutations and 2‐HG on the tumor microenvironment is a highly active area of research. We analyzed published clinical datasets^[^ ^25^ ^]^ and performed gene set enrichment analysis (GSEA) to identify significantly altered gene sets. We found that gene sets associated with mitotic activities are significantly enriched in the IDHwt tumors (**Figure** **1**A), which may correlate with their relatively faster progression. Notably, we found a significant enrichment of adaptive immune responses, particularly lymphocyte‐related immunity, in IDHwt tumors (Figure S1A, Supporting Information). Estimation of the intratumoral immune composition with the tumor immune estimation resource (TIMER) (https://cistrome.shinyapps.io/timer/) indicated that the infiltration of B cells, CD8+ T cells, macrophages, neutrophils, and dendritic cells is significantly reduced by the presence of IDH mutations (Figure S1B, Supporting Information). This is consistent with the observation in the IDH‐isogenic mouse model, where tumor infiltrating CD4+ and CD8+ cells are reduced by the expression of IDHmut.^[^ ^26^ ^]^ Furthermore, gene sets related to intratumoral vasculature formation displayed distinct expression patterns between IDHwt and IDHmut tumors (Figure 1A). Together, these results indicate that IDH mutations dictate a unique immune and vascular microenvironment in gliomas.

We further analyzed the expression of previously defined 26 angiogenesis associated genes (VCAN, POSTN, FSTL1, LRPAP1, STC1, VEGFA, THBD, TNFRSF21, COL5A2, ITGAV, APP, JAG1, COL3A1, SPP1, NRP1, OLR1, PDGFA, PTK2, VAV2, S100A4, MSX1, TIMP1, PRG2, JAG2, LUM, CXCL6)^[^ ^27^ ^]^ in IDHmut tumors. The average expression levels of these genes are significantly lower in IDHmut tumors (Figure 1B). Survival analyses comparing tumors with high versus low expression of this 26‐gene signature suggest that its expression is negatively associated with prognosis (Figure 1C). These findings indicate that levels of angiogenesis indeed represent predictive value for prognosis, and highlight the importance of considering anti‐angiogenic strategies.

In clinical settings, angiogenesis‐targeting strategies for gliomas have yielded mixed results, particularly evident in VEGF‐targeting clinical trials where these agents failed to extend patient survival.^[^ ^28^, ^29^, ^30^ ^]^ However, these trials primarily involved patient cohorts with glioblastomas, which are predominantly IDHwt tumors by the nature of the disease. The potential effectiveness of specific angiogenesis‐targeting approaches for managing IDHmut gliomas remains underexplored. Given the distinctly shaped microenvironment in IDHmut tumors, we were prompted to investigate this concept further in the laboratory.

### 2.2 Hypoxia Amplifies IDHmut Induction of 2‐HG and Suppresses DNA Damage Repair Synergistically

Tumor angiogenesis and hypoxia are closely interconnected processes. As tumors grow, their rapid proliferation often outpaces the development of an adequate blood supply, leading to regions of low oxygen levels. Hypoxia, in turn, activates adaptive signaling pathways, including the stabilization of HIF‐1α, which promotes the expression of pro‐angiogenic factors such as VEGF. This feedback loop drives the formation of new blood vessels to restore oxygen delivery, while also contributing to a heterogeneous and often dysfunctional tumor vasculature. Therefore, the hypoxic tumor microenvironment not only results from insufficient angiogenesis but also actively stimulates further vascularization, linking these two processes in a dynamic regulatory network.

Under hypoxic conditions, mammalian cells produce L‐2‐HG that is sufficient to induce a characteristic hypermethylation phenotype of histones similar to that observed in IDHmut contexts.^[^ ^31^ ^]^ L‐2‐HG promotes HIF‐1α stabilization and sustains tumor growth, while the role of D‐2‐HG on HIF‐1α stability is diverse in a context‐dependent manner.^[^ ^32^, ^33^ ^]^ In glioma, understanding whether and how IDHmut affects the level of HIF‐1α is important to comprehend how oncogenic IDH mutations promote tumor adaptation to oxygen and nutrition limitation. We determined HIF‐1α levels in an immortalized human astrocyte (IHA) cell line where expression of IDHmut mimicked the glioma CpG island methylator phenotype (G‐CIMP) observed in most IDH mutant gliomas.^[^ ^10^ ^]^ Hypoxia rapidly increased the level of HIF‐1α, which reached a peak at 24 h (Figure 1D). Yet, the HIF‐1α level did not seem to discriminate the mutation status of IDH1 (empty vector (EV) vs IDHmut, 24 h), with the pattern of increase in protein level displayed little difference (Figure 1D). Under aerobic conditions, however, HIF‐1α levels were significantly higher in IHA with IDHmut expression than in EV control. Moreover, mRNA levels of immediate HIF‐1α targets, including LDHA, PDK1, PGK1, SLC2A1, and VEGFA, are significantly higher in IDHmut IHA under normoxia (Figure 1E). Thus, ectopic HIF‐1α expression may subject IDHmut cells to pseudohypoxia, a process that increases the NADH/NAD+ ratio, and may constitute the basis of hypersensitivity to NAD+ deprivation that has been observed in a glioma‐related context.^[^ ^34^ ^]^ Although previous studies indicated that IDHmut was associated with a higher level of hypoxic response in LGG patients,^[^ ^35^ ^]^ our study did not observe synergistic effects with IDHmut on HIF‐1α stabilization under hypoxic conditions.

Under hypoxia, the cell shifts its glucose metabolism toward increased lactate production, which subsequently increases the cellular concentration of α‐ketoglutarate due to the need for citrate synthesis.^[^ ^36^ ^]^ Concurrent IDHmut expression, therefore, may catalyze the conversion of this excessive α‐KG into 2‐HG under hypoxic conditions and result in further 2‐HG accumulation in a synergistic manner. To test this, we measured the increase in cellular 2‐HG concentration in IHA cells under IDHmut expression and hypoxia with liquid chromatography‐mass spectrometry (LC‐MS). The results showed that, consistent with previous findings,^[^ ^31^ ^]^ hypoxia promotes 2‐HG production (Figure 1F). While IDHmut expression modestly increases 2‐HG level under normoxia, it markedly increases 2‐HG levels under hypoxia in a synergistic, rather than additive manner (> 250 folds in IDHmut + hypoxia vs ≈125 folds in IDHmut, Figure 1F). Previous studies have shown that 2‐HG suppresses HR efficiency in a KDM4 lysine demethylase‐dependent manner.^[^ ^37^ ^]^ Therefore, the synergy between IDHmut and hypoxia in driving 2‐HG accumulation likely renders cells hypersensitive to agents that disrupt DNA damage repair. To address this, we first examined the presence and levels of γ‐H_2_AX foci that mark unrepaired DNA breaks in IHA cells. The results showed that while hypoxia or IDHmut expression moderately induces accumulation of γ‐H_2_AX foci, their combination markedly suppresses the resolving of γ‐H_2_AX foci (Figure 1G,H). To directly assess homologous recombination (HR) repair activity, we performed immunofluorescence staining for RAD51 foci formation (Figure S2A, Supporting Information). While γ‐H_2_AX foci mark DNA double‐strand breaks, RAD51 recruitment to these sites provides a functional readout of HR capacity, as RAD51 nucleoprotein filaments are essential for strand invasion and repair. Consistent with this, the presence and quantification of RAD51 foci after DNA damage are widely used as a surrogate for HR efficiency. In our experiments, we observed robust RAD51 foci induction by temozolomide (TMZ) in both IDHwt and IDHmut cells (Figure S2A, Supporting Information). However, IDHmut cells displayed dampened RAD51 foci formation to a greater extent compared with IDHwt cells under hypoxia (Figure S2A, Supporting Information). Consistent with this, comet assays showed similar synergy, with significantly longer comet tails observed in IHA under IDHmut + hypoxia compared to either condition alone (Figure 1I,J). Meanwhile, to test whether the synergy was tissue‐specific, we determined the IDHmut‐hypoxia impairment in DNA damage in an intrahepatic cholangiocarcinoma cell line (HUCCT1). We observed similar IDHmut‐hypoxia synergy in γ‐H_2_AX foci (Figure S2B, Supporting Information) and comet tail moments (Figure S2C, Supporting Information). Thus, IDHmut cells displayed dampened DDR capability under hypoxia.

### 2.3 Hypoxia Induces Apoptosis in IDHmut Cells Under PARP Inhibition

Having established the cooperativity of IDHmut and hypoxia in driving DDR defects, we aimed to determine their combinatory effect on inducing cell death. Hypoxia induces cell death marked by propidium iodide, whereas expression of IDHmut did not change propidium iodide labeling positivity (Figure 1K; Figure S3A, Supporting Information). The PARP inhibitor veliparib, which shows killing specificity toward IDHmut cells,^[^ ^18^ ^]^ also demonstrated this specificity in our study (Figure 1K; Figure S3A, Supporting Information).

However, the combination of veliparib and hypoxia significantly increased cell death compared to either treatment alone in IDHmut IHA cells, an effect not observed in WT IHA cells under hypoxia (Figure 1K; Figure S3A, Supporting Information). Additionally, clonogenic assays with IDHmut or EV IHA showed that veliparib treatment under hypoxia cooperatively suppressed the clonogenicity of IDHmut IHA and HUCCT1 to a significantly greater extent (Figure 1L; Figure S3B,C, Supporting Information).

### 2.4 A Regimen Combining Veliparib and Lenvatinib Limits the Growth of IDHmut Brain Tumors

**Figure 2:**
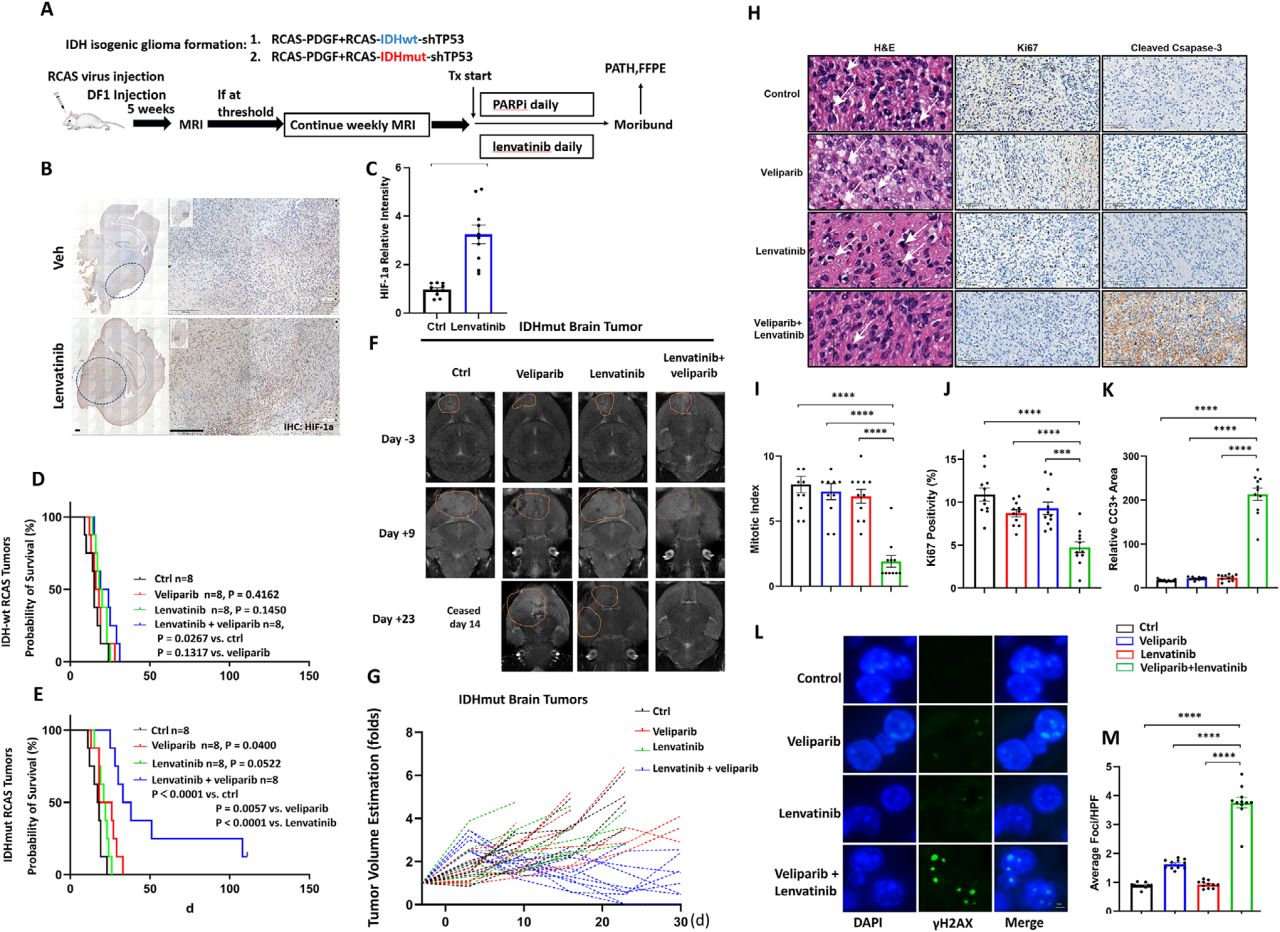
A combination of veliparib and lenvatinib significantly prolonged the survival of mice bearing IDH1mut glioma. A) Schematic outline of treatments. Mice received DF‐1 inoculation, constitutively producing RCAS viruses. All mice received weekly MRI scans post‐inoculation. Mice with established tumors over a defined size threshold were randomized to vehicle, veliparib (25 mg kg^−1^d^−1^), lenvatinib (10 mg kg^−1^d^−1^), or veliparib + lenvatinib arms. TX, treatment; PATH, pathologic analysis. FFPE: formalin‐fixed and paraffin embedded. B) Representative images (N = 3) of immunohistochemical staining of HIF‐1α. Dotted circles indicate the locations and areas of brain tumors. Scale bar: 200 µm. C) Relative staining positivity of 10 independent fields was quantified and plotted as mean ± SEM. N = 10. D) Kaplan‐Meier analysis of mice bearing IDH1‐wt glioma, starting from the day corresponding treatment initiated. *P* values were determined by the log‐rank (Mantel‐Cox) test. The number of animals per group (n) is represented on the plot. E) Kaplan‐Meier analysis of mice bearing IDH1mut glioma. The number of animals per group (n) is represented on the plot. F) Representative images of MRI scans of IDHmut brain tumors at days ‐3, 9, and 23. G) Growth curves of individual brain tumors as estimated by MRI at the largest slices. H) Mice bearing IDH1mut glioma were sacrificed at day 12 post treatment initiation. H&E staining (left panels), immunohistochemistry staining of Ki67 (middle panels) and cleaved caspase 3 (right panels) was performed on FFPE tumor sections. Scale bar represents 10 µm. I, J and K) Relative intensity of mitotic index, Ki67 positivity and cleaved caspase‐3 (CC3) was quantified with ten independent 20× fields and plotted on bar graphs as mean ± SEM. N = 11. L) Immunofluorescent staining of γ‐H_2_AX in FFPE sections of mice brain tumor tissues at day 12 after treatment. Scale bar represents 10 µm. M) Quantification of (E), performed by counting the average foci in the nuclei. HFP: high power field. N = 11.^*^ *p* < 0.05, ^**^ *p* < 0.01, ^***^ *p* < 0.001, ^****^ *p* < 0.0001, ns: not significant.

The cooperativity of PARP inhibitors and hypoxia in IHA and HUCCT1 cells prompted us to determine their in vivo effectiveness in treating IDHmut tumors. Since angiogenesis inhibition is known to promote intratumoral hypoxia,^[^ ^38^ ^]^ we hypothesized that combining an anti‐angiogenic agent with PARP inhibition might enhance tumor sensitivity by exploiting this hypoxic microenvironment. Indeed, combination treatments of PARP inhibitors and antiangiogenic agents have been proposed, with some clinical trials following this strategy being implemented.^[^ ^39^ ^]^ Yet, most of these studies primarily tested efficacy in BRCA‐deficient patients, leaving out BRCA‐proficient patients with high BRCAness features that might potentially benefit from this strategy. Given the established sensitivity of IDHmut tumors to single‐agent PARP inhibition, it is particularly important to evaluate the efficacy of this combination strategy in this context. For this purpose, we used the RCAS‐driven brain tumor model with isogenic expression of either WT or R132H mutant IDH1.^[^ ^26^ ^]^ The Nestin‐TVA mice express the TVA receptor under the Nestin promoter, allowing selective infection of neural progenitor cells by RCAS viruses produced from DF1 cells. Stereotactic injection of DF1 cells ensures localized production of RCAS viruses in the brain, enabling efficient and spatially controlled gene delivery to neural progenitors. This approach allows us to model glioma initiation and progression in a physiologically relevant manner. For drug efficacy tests, homozygous Nestin‐TVA mice received stereotactic DF1 cell injections, and the brain tumors were allowed to grow for 5 weeks before MR imaging was performed to determine the tumor formation and size. The tumor‐bearing mice were then stratified based on tumor size and randomized into four treatment groups: vehicle control, veliparib, lenvatinib, and combination of the two (**Figure** **2**A).

Brain tumors, particularly gliomas, are known for their abnormal vasculature, which results in heterogeneous oxygen distribution. Anti‐angiogenic therapies exacerbate this issue by impeding neovascularization, leading to regions of severe hypoxia.^[^ ^40^ ^]^ However, previous studies have proposed that the therapeutic benefit of anti‐angiogenic agents may instead arise from vascular normalization, which alleviates hypoxia and improves tumor perfusion.^[^ ^41^, ^42^, ^43^, ^44^ ^]^ Thus, to investigate whether lenvatinib treatment increases hypoxia within tumors, we assessed the levels of HIF‐1α with immunohistochemical staining. The results (Figure 2B,C) showed that lenvatinib treatment markedly increased hypoxia levels within the tumors, providing a mechanistic basis for the combinational use of drugs in our study.

In the IDH‐wt context, veliparib and lenvatinib alone showed little efficacy in the survival of tumor‐bearing mice (Figure 2D). While the combination significantly prolonged survival (combo vs ctrl, *p* = 0.0267), it only slightly extended median survival and did not significantly improve survival compared to single treatments, suggesting an additive effect rather than synergy. By contrast, in the IDHmut background, lenvatinib + veliparib treatment showed longer survival time compared to the vehicle group (combination 39 d vs ctrl 18 d, Figure 2E). Notably, while PARPi alone displayed moderate efficacy (*p* = 0.0400), lenvatinib alone only displayed marginal benefit in survival that is not statistically significant (*p* = 0.0522). Therefore, at least in our model, an anti‐angiogenic drug as a single agent failed to inhibit tumor growth regardless of IDH mutational status. The follow‐up on tumor sizes assessed by MR imaging indicated that the combination treatment achieved pronounced tumor regression in some cases (Figure 2F,G). Together, these data established preclinical evidence strongly supporting the therapeutic potential of combining PARPi and anti‐angiogenic agents in IDHmut gliomas.

### 2.5 Combination Treatment Promotes Significant Changes in Proliferation, Apoptosis, and DDR in IDHmut Brain Tumors

Having confirmed the cooperativity of lenvatinib and veliparib, we sought to investigate the underlying mechanisms that drive this synergism. While the rationale for combining PARP inhibition with anti‐angiogenic therapy has been supported by experimental data and evaluated in clinical settings, the precise molecular crosstalk between DNA damage repair pathways, hypoxic signaling, and angiogenesis remains poorly defined. To investigate these mechanisms, we analyzed the tumors following lenvatinib, veliparib, or lenvatinib + veliparib treatment. Since end stage tumors collected at moribund may not accurately reflect drug responses, we set up a new 4‐armed group with identical treatment regimens, sacrificed mice, and collected tumor samples at day 12 post‐treatment initiation.

We next determined proliferation, apoptosis, and DDR markers with H&E staining, immunohistochemistry (IHC) labeling of Ki67 and cleaved caspase‐3, and immunofluorescence (IF) labeling of γ‐H_2_AX. The results showed that tumor sections in the combination group displayed significantly lower levels of mitotic activities as determined by a pathologist (Y.L.) (Figure 2H,I), lower levels of Ki67 labeling positivity (Figure 2H,J), higher levels of cleaved caspase‐3 (Figure 2H,K), and higher levels of γ‐H_2_AX (Figure 2L,M). These data strongly support the role of lenvatinib + veliparib combination in limiting tumor growth, consistent with the survival analysis findings.

### 2.6 Transcriptional Profiling of Tumor Samples Identified Key Mediators for Single Agent Resistance

**Figure 3:**
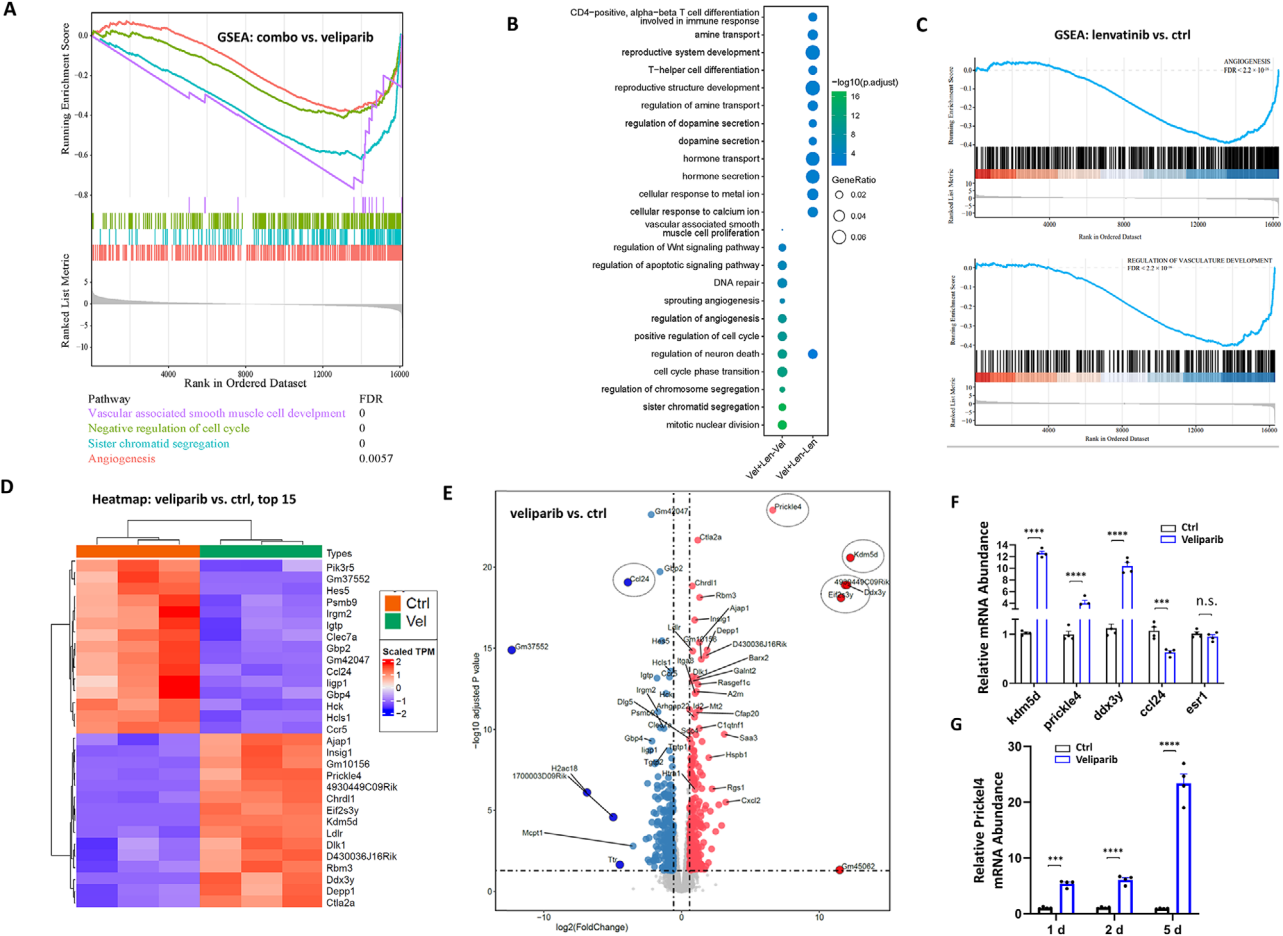
Transcriptome studies revealed key mediators for the drug combination. A) RNA sequencing was performed in IDH1mut glioma tissue post drug treatments. Gene Set Enrichment Analyses (GSEA) showed the pathways associated with mitotic activity have been changed in veliparib + lenvatinib treatment comparing to veliparib. B) Gene ratios and adjusted *p* values were scaled. Comparisons performed were: left: veliparib + lenvatinib (combo) versus veliparib; right: veliparib + lenvatinib (combo) versus lenvatinib. C) GSEA comparing Lenvatinib versus control demonstrated significant alterations in the “Angiogenesis” and “Regulation of vasculature development” gene sets. D) Heatmap of top 15 genes with significantly altered expression post veliparib treatment. E) Volcano plot for differential expression of all genes comparing veliparib versus vehicle (ctrl) treated tumors. F) RCAS‐IDHmut tumor samples treated with veliparib were analyzed by qRT‐PCR for mRNA expression of *kdm5d*, *prickle4*, *ddx3y*, *ccl24* and *esr1*. The average mRNA levels were represented on bar graphs as mean ± SEM. N = 4. G) IHA treated with veliparib at 1, 2 and 5d were analyzed by qRT‐PCR for mRNA levels of *prickle4*. N = 4. ^*^ *p* < 0.05, ^**^ *p* < 0.01, ^***^ *p* < 0.001, ^****^ *p* < 0.0001, ns: not significant.

As indicated above, the combination of veliparib and lenvatinib displayed robust tumor regressive activity that neither drug alone has achieved. While our in vitro studies established the cooperativity between hypoxia and PARPi in inducing DDR defects, the in vivo efficacy of this combination raised new questions about resistance mechanisms in the tumor microenvironment. We reasoned that resistance to a single agent may be linked to molecular changes post one drug treatment that render tumor cells sensitive to another, rather than a simple combinatory effect of drugs. Alternatively, resistance mechanisms triggered by one agent may be counteracted by the complementary activity of the other. These changes can be broadly captured by genomic studies. To determine this, we subjected fresh tumor samples, collected 12 days post treatment, to transcriptional profiling with bulk RNA sequencing. Notably, we observed significant changes associated with proliferation in the combination treatment. Gene ontology (GO) and gene set enrichment analyses (GSEA) showed significant alterations in the gene sets related to cell division, including *nuclear division, sister chromatid segregation, and mitotic nuclear division* (**Figure** **3**A, combo vs veliparib, Figure S4A,B, Supporting Information, combo vs ctrl), providing further evidence for the efficacy of the drug combination.

It is also evident in the GO analyses where a single agent alone promoted pathway changes that potentially connect to the resistant mechanisms. In tumors treated with veliparib + lenvatinib compared to lenvatinib, we observed significant changes in pathways that regulate cytokine release and the immune system, such as inflammatory response pathways, including “*T cell differentiation”* and “*T‐helper differentiation”* (Figure 3B, combo vs lenvatinib). This is consistent in the veliparib versus ctrl comparison, where regulatory pathways of *“regulation of inflammatory response”, “regulation of T cell activation”*, and *“response to interferon‐gamma”* displayed significant changes (Figure S5, Supporting Information, Ctrl‐Vel). Conversely, lenvatinib treatment indicated pathway changes that were, to our knowledge, less instructive regarding resistant mechanisms (Figure S5, Supporting Information, Ctrl‐Len and ctrl‐Vel + Len), other than modification of vascular functions, as supported by negative enrichment of the “*angiogenesis*” and “*regulation of vasculature development*” gene sets (Figure 3C).

Next, we examined the differentially expressed genes in veliparib treated tumors compared to controls. Veliparib treatment led to widespread changes in transcription profile (Figure 3D,E; Figure S6, Supporting Information). For validation, we subjected tumor RNA samples to reverse transcription PCR (RT‐PCR) for the determination of the expression differences in *kdm5d, prickle4, ddx3y*, and *ccl24*. Consistent with RNAseq data, the expression of *kdm5d, prickle4, and ddx3y* was significantly increased, while *ccl24* expression was significantly decreased post veliparib treatment (Figure 3F). As an internal control, the expression level of *esr1* did not change in RNAseq nor did it in RT‐PCR results (Figure 3F).

### 2.7 Transcriptional Changes Post Veliparib Treatment Facilitate Tumor Vascularization

**Figure 4:**
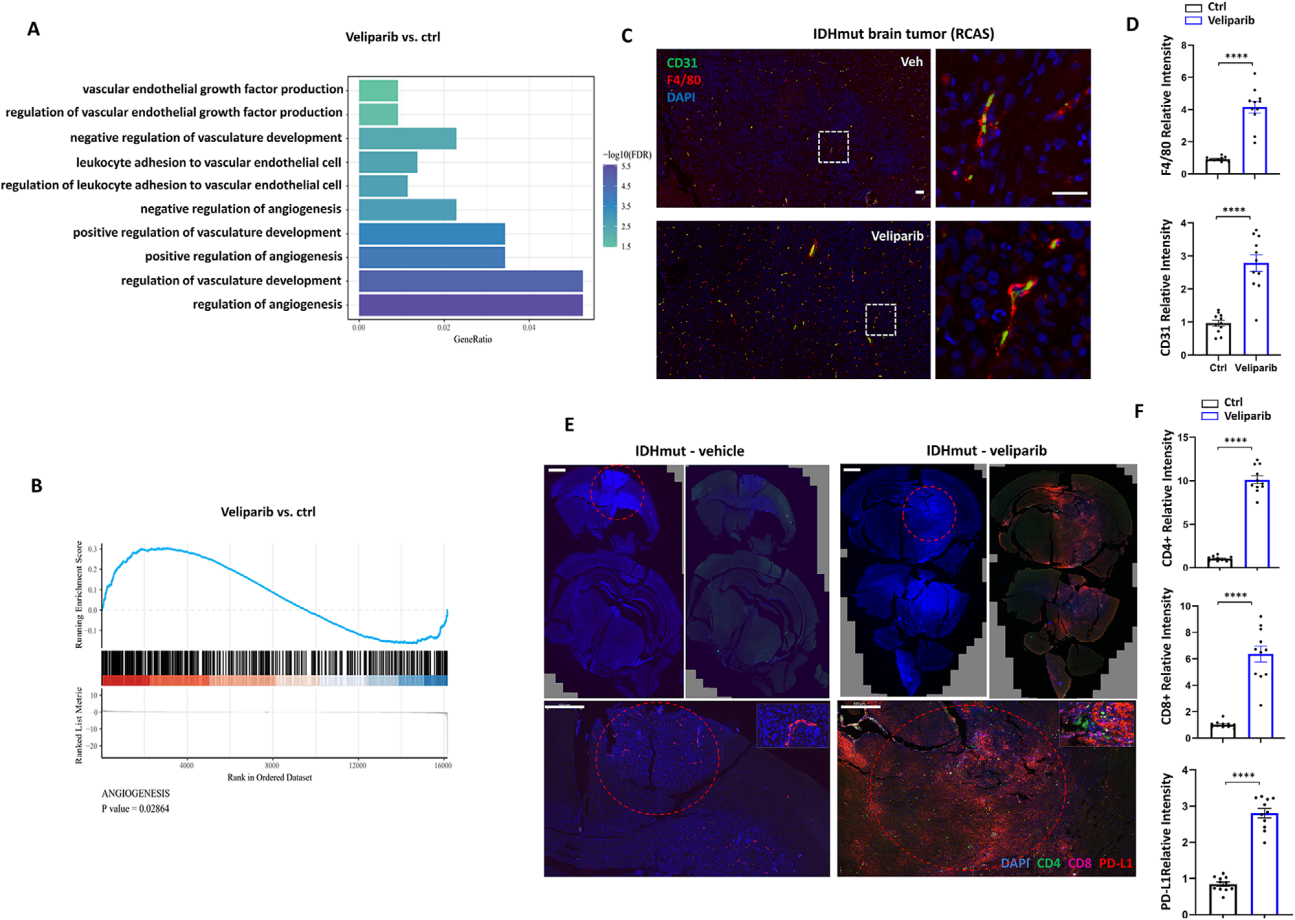
The tumor microenvironment was altered by veliparib treatment towards pro‐angiogenic. A) Gene ontology analysis comparing veliparib and control treated IDHmut tumors, showing significant alterations in angiogenesis regulation pathways. B) GSEA analysis comparing veliparib and control treated IDHmut tumors, showing significant enrichment in the “angiogenesis” gene set. C) Mice bearing IDH1mut glioma were sacrificed at day 12 post treatment initiation with vehicle or veliparib, and tumor tissues were fixed and paraffin sections were made. Immunofluorescent staining of CD31 and F4/80 was performed on FFPE tumor sections. Scale bar represent 50 µm. D) Relative labeling intensity of F4/80 and CD31 in 10 independent fields was quantified and plotted on bar graphs as mean ± SEM. N = 11. E) Mice bearing IDH1mut glioma were sacrificed at day 12 after treatment with vehicle or veliparib. Immunofluorescent staining of DAPI, CD4, CD8 and PD‐L1 were performed. Red circles indicate tumor areas. Scale bar represent 500 µm. F) Relative intensity of CD4, CD8 and PD‐L1 of 10 high power fields in vehicle and veliparib were quantified and plotted. N = 11. ^*^ *p* < 0.05, ^**^ *p* < 0.01, ^***^ *p* < 0.001, ^****^ *p* < 0.0001, ns: not significant.

Prickle4 (prickle planar cell polarity protein 4) participates in a highly conserved, Wnt‐driven signaling cascade that regulates planar cell polarity (PCP). To validate the RNA‐seq results, we conducted a time course study showing that in cultured IHA, veliparib treatment led to robust induction of Prickle4 expression (Figure 3G). Thus, we speculated that increased Prickle4 may promote brain tumor angiogenesis that forms the resistance mechanism to PARPi. In supporting this, in a targeted analysis with angiogenesis‐related gene signatures, we found that gene sets including “*regulation of angiogenesis*”, “*regulation of vasculature development*,” and *“regulation of vascular endothelial growth factor production*” were significantly altered by veliparib treatment (**Figure** **4**A). Also, GSEA analysis with the “*angiogenesis*” gene set demonstrated significant enrichment (Figure 4B). To determine the level of angiogenesis following PARPi, we stained RCAS‐IDHmut brain tumor samples with the endothelial marker CD31 and the macrophage/microglia marker F4/80 (Figure 4C,D). Typical endothelial cells surrounded by macrophages were evident, and the total level of CD31 was significantly higher in veliparib treated tumors (Figure 4C,D). These results strongly support that veliparib treatment induced a pro‐angiogenic microenvironment that may contribute to drug resistance.

Interestingly, the levels of lymphocyte infiltration and PD‐L1 expression also increased following veliparib treatment (Figure 4E,F). This observation was consistent with gene ontology studies derived from RNA‐seq, where multiple immune stimulatory pathways were identified, such as *response to interferon gamma, regulation of T cell activation*, and *regulation of inflammatory response*. Notably, veliparib also induces PD‐L1 increase in RCAS‐IDHwt tumors (Figure S7, Supporting Information). However, T cell infiltration was to a lesser extent, in contrast to the observation in the IDHmut tumors (Figure S7, Supporting Information).

### 2.8 Prickle4 Expression is Associated with Malignant Cells, Inducing Angiogenesis, And Suppression of Prickle4 Reversed Resistance to Parpi

**Figure 5:**
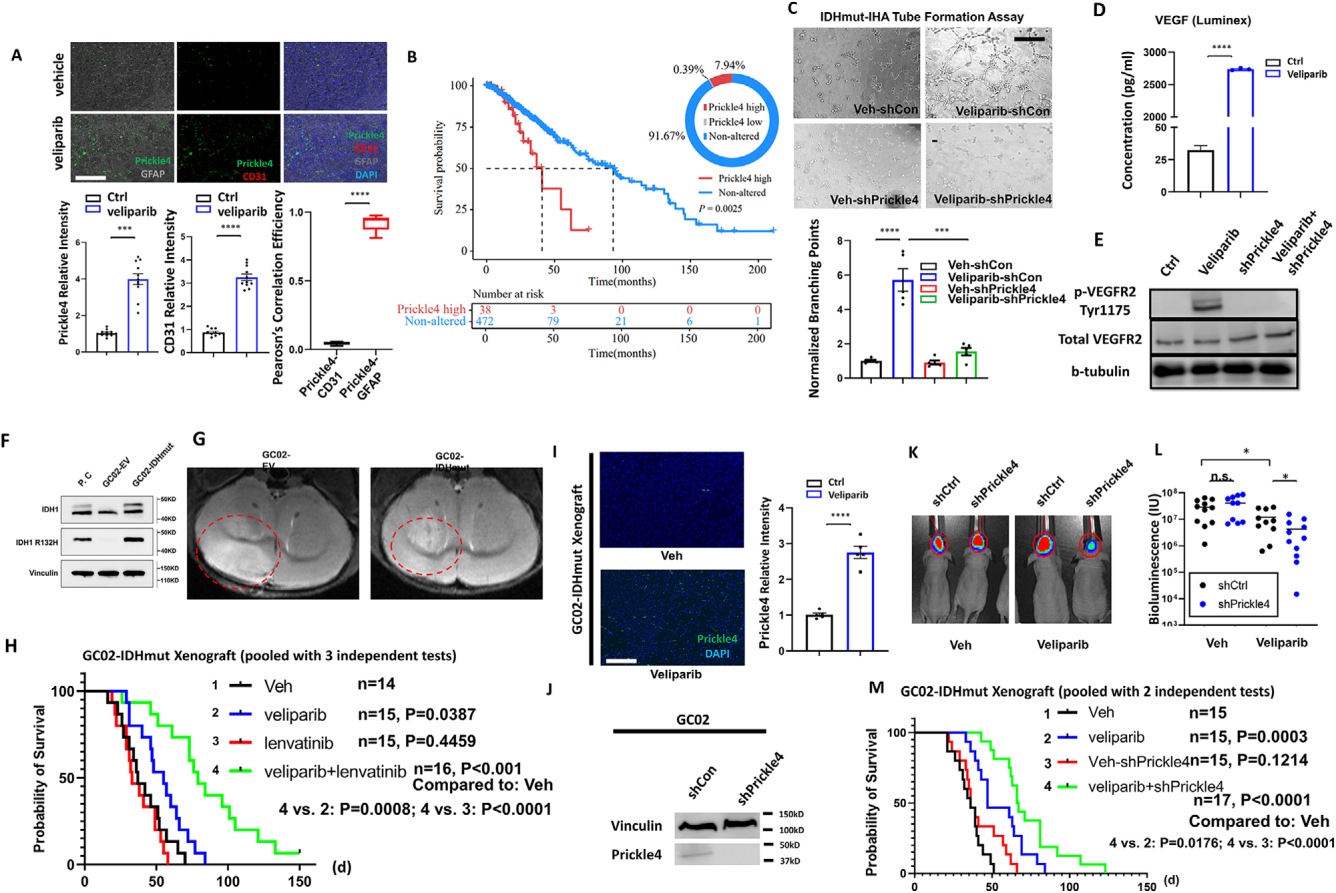
Prickle4 suppression is effective to overcome veliparib resistance in IDHmut brain tumors. A) immunofluorescent staining of Prickle4 (green), CD31(red) and GFAP (gray) with veliparib or vehicle treated RCAS‐IDHmut tumors. Scale bar: 100 µm. Quantification of Prickle4 and CD31 labeling positivity of 10 independent fields were plotted onto bar graphs as mean ± SEM. Pearson's correlation coefficient for colocalization of Prickle4‐CD31 and Prickle4‐GFAP was plotted as mean ± SEM. N = 10.B) Percentage of patient tumors with Prickle4 high expression and Kaplan‐Meier plot of overall survival (OS) comparing Prickle4‐high and non‐altered groups. P value was calculated by log‐rank (Mantel‐Cox) test. C) Representative images of tube formation by HUVEC cells at 6 h treated with culture supernatant from IHA. Scale bar: 500 µm. Quantification of branching point fold change was represented on bar graphs for each condition as mean ± SEM. N = 5. D) Concentration (pg ml^−1^) of VEGF in the culture medium treated with vehicle (ctrl) or veliparib, determined by Luminex 200. N = 3. E) Phosphorylation levels of VEGFR2 (Tyr 1175) and total levels of VEGFR2 in HUVEC cells were determined by Western blots. Beta‐tubulin was taken as internal control. N = 3. F) Western blots determining the level of IDH1wt (upper), IDH1R132H (middle) and Vinculin (loading control, bottom). P.C: positive control (GL261‐IDHmut lysate with known IDHmut expression). G) Representative MRI scans of GC02‐EV (left) and GC02‐IDHmut (right). H) Kaplan‐Meier analysis of mice bearing GC02‐IDHmut xenograft on vehicle, veliparib, lenvatinib and the combination treatment. *P* values were determined by log‐rank (Mantel‐Cox) test. The number of animals per group (n) is represented on the plot. I) Immunofluorescent staining of Prickle4 in veliparib/vehicle treated GC02 xenografts. Scale bar: 100 µm. Relative intensity of Prickle4 labeling positivity was quantified and plotted as mean ± SEM. N = 5. J) Western blots of Prickle4 and Vinculin (loading control) were performed with shRNA transduced GC02 cells. Relative intensity of Prickle4 protein abundance was quantified and plotted on bar graphs as means ± SEM. K) Representative bioluminescent images of GC02 xenograft on day 28 post veliparib treatment. L) Average BLI signal intensities were quantified and represented as mean ± SEM. N = 11. M) Kaplan‐Meier analysis of mice bearing GC02‐IDHmut xenograft with Prickle4 knockdown on a veliparib treatment. *P* values were determined by log‐rank (Mantel‐Cox) test and represented. The number of animals per group (n) is represented on the plot. ^*^ *p* < 0.05, ^**^ *p* < 0.01, ^***^ *p* < 0.001, ^****^ *p* < 0.0001, ns: not significant.

To determine whether Prickle4 induction directly influences endothelial cells or instead functions in an indirect, tumor cell‐derived paracrine manner, we performed immunofluorescent staining for Prickle4, CD31, and GFAP (**Figure** **5**A). Consistent with previous results, the levels of Prickle4 and CD31 increased following veliparib treatment (Figure 5A). Notably, Prickle4 expression did not colocalize with CD31, but was instead associated with the tumor marker GFAP (Pearson's correlation analyses, Figure 5A). This suggests that veliparib may induce elevated Prickle4 expression in the tumor cells, thereby stimulating pro‐angiogenic factors facilitating tumor vascularization. Indeed, a significant portion of LGG tumors (≈8%) displayed high Prickle4 expression (FC > 1.5) (Figure 5B). Survival analysis of LGG patients further indicated that high Prickle4 expression is associated with significantly poorer clinical outcomes (Figure 5B). Together, these results suggest that veliparib‐induced Prickle4 expression in tumor cells may contribute to tumor progression in our model.

To further investigate whether Prickle4 expression enhances the angiogenic potential of endothelial cells, we performed tube formation assays, where culture supernatant collected from IHA was added to HUVEC cells to assess tube formation efficiency (Figure 5C). The results showed that veliparib significantly enhanced the ability of culture supernatant to induce in vitro angiogenesis (Figure 5C), while this induction is abolished when shRNA against Prickle4 was introduced. To further assess the changes in cytokine secretion profiles, we performed flow cytometry‐based Luminex 200 assay, determining the concentration of a panel of 48 cytokines in the culture media. The results indicated that the concentration of VEGF was significantly increased by veliparib treatment (Figure 5D). Consistently, the co‐culture of these media with HUVEC cells showed activation of the angiogenesis pathway mediated by VEGFR2 phosphorylation, whereas such activation was abolished by knockdown of Prickle4 (Figure 5E). Rescue of Prickle4 expression in these Prickle4 knockdown cells effectively restored VEGFR2 phosphorylation in co‐cultured HUVEC cells (Figure S8A, Supporting Information), addressing the essential role of Prickle4 in mediating angiogenesis. Furthermore, to substantiate the role of VEGF pathway activation in PARPi‐induced angiogenesis, we treated HUVEC cells with lenvatinib in the co‐culture setting and observed decreased VEGFR2 phosphorylation at 1, 5, and 10 µm (Figure S8B, Supporting Information). Similarly, neutralization of VEGF in the conditioned medium by bevacizumab effectively blocked VEGFR2 phosphorylation (Figure S8C, Supporting Information). Furthermore, expression data in the clinical samples indicated a significant positive correlation between Prickle4 and VEGFA expression, providing supportive proof for the involvement of VEGF signaling and Prickle4 expression (Figure S8D, Supporting Information). Of note, while our findings establish Prickle4 as a necessary mediator of PARPi‐induced angiogenesis, future in vivo studies combining Prickle4 knockdown with anti‐angiogenic therapy will be important to more definitively establish whether Prickle4 lies epistatically upstream of VEGF signaling in mediating resistance.

Next, we aimed to epistatically identify the necessary role of Prickle4 in mediating tumor angiogenesis and PARPi resistance in the animal model. We constructed an RCAS‐shPrickle4 vector but failed to induce Prickle4 knockdown in the RCAS‐driven tumors. We reasoned that the entry or replication efficiency of the shPrickle4 virus may have been limited by the presence of other RCAS viruses in the model. Upon further investigation into the literature and discussion with colleagues, we found that the successful delivery of additional shRNA constructs in this system has rarely been achieved. The more prevalent strategy involves crossing the animals with strains carrying the desired genotypes. However, a Prickle4 knockout or floxed mouse model is not immediately available, preventing us from verifying the role of Prickle4 using this approach. Due to the limitations of the RCAS‐based gene silencing strategy, we in‐house generated our glioma stem‐like cell line (GC02) from a patient who was 71 years old at diagnosis (2021), carrying a WHO grade IV glioblastoma in the right occipital lobe. The resected tumor specimen was stained negatively for IDH1R132H and positively for ATRX (Figure S9A, Supporting Information). The GC02 cell line was maintained in stem cell culture conditions and grew in neurosphere suspensions (Figure S9B, Supporting Information). We ectopically expressed IDH1R132H (IDHmut) or the empty vector (EV) control in this cell line and validated IDHmut expression with Western blots (Figure 5F). These isogenic cell lines were capable of generating orthotopic xenografts in NOD.Cg‐Prkdc^scid^Il2rg^em1Smoc^ (NSG) mice (Figure 5G). The xenograft tumors displayed infiltrative growth into surrounding brain tissues (Figure S9C, Supporting Information). This model presents greater clinical relevance and serves as a complementary approach to the RCAS‐TVA model by enabling additional mechanistic studies and genetic manipulation. We also introduced a luciferase reporter (HIV‐Luc‐ZsGreen), enabling us to monitor bioluminescent imaging (BLI) following xenotransplantation. The genetic background of the GC02‐IDHmut xenograft model is apparently different from the RCAS‐TVA model. Therefore, we also tested the cooperativity of veliparib and lenvatinib with the GC02‐IDHmut model. Similarly with previous studies,^[^ ^14^, ^18^, ^37^ ^]^ veliparib inhibited tumor growth in this GC02‐IDHmut GSC line (Figure 5H). Moreover, the results supported that the combined use of drugs significantly extended the survival time of IDHmut tumor‐bearing mice (Figure 5H), similar to what was observed in the RCAS‐TVA model. Staining of tumor specimen indicated that treatment of veliparib also induced expression of Prickle4 in GC02 xenografts (Figure 5I). In contrast, all these regimens failed to improve the survival of mice bearing GC02‐IDHwt tumors (Figure S10, Supporting Information).

Next, we investigated whether Prickle4 expression is necessary for PARPi resistance. For this purpose, we delivered the Prickle4 shRNA construct into GC02‐IDHmut and confirmed successful Prickle4 knockdown by Western blots (Figure 5J). BLI results (Figure 5K,L) from these GC02‐IDHmut xenograft tumors on day 28 showed that Prickle4 knockdown did not affect tumor growth (Figure 5K,L), nor did it affect the survival of mice (Figure 5M) without veliparib treatment. Yet, under veliparib treatment, Prickle4 knockdown further inhibits tumor growth and extends survival (Figure 5K–M).

**Figure 6:**
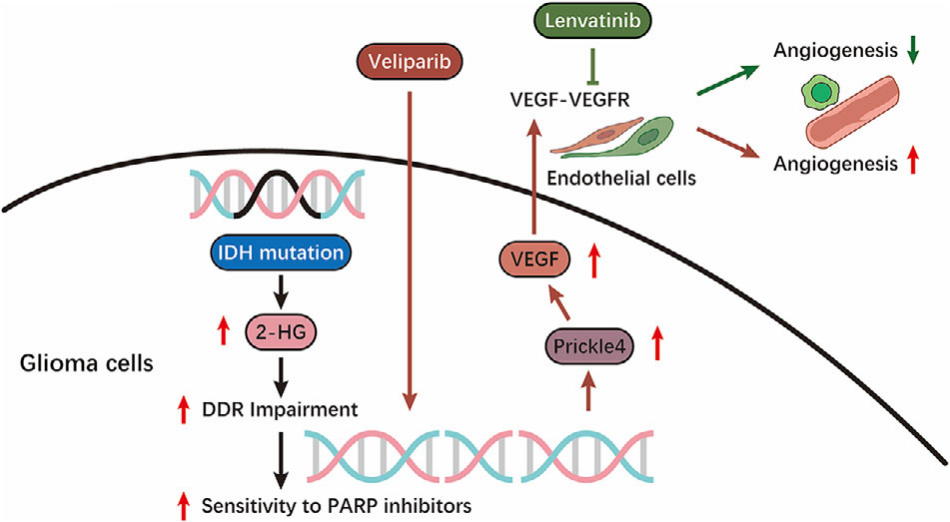
Proposed mechanistic model linking IDH mutations, PARP inhibition, Prickle4, and angiogenesis in glioma. IDH mutations increase 2‐HG production, leading to DDR pathway impairment and thereby sensitizing glioma cells to PARP inhibition. Treatment with veliparib exacerbates DDR stress but also induces upregulation of Prickle4. Elevated Prickle4 promotes VEGF expression, which stimulates endothelial cells via VEGF‐VEGFR signaling, resulting in enhanced angiogenesis and therapy PARPi resistance. Pharmacological blockade of VEGF‐VEGFR signaling with lenvatinib mitigates this pro‐angiogenic response, restoring the efficacy of PARPi treatment. Together, the model highlights Prickle4 as a key mediator of PARPi‐induced angiogenesis and suggests that combined targeting of PARP activity and angiogenic signaling could improve therapeutic outcomes in glioma.

Thus, we identified Prickle4 as a key factor that is induced by PARPi and promotes tumor angiogenesis, which underlies the therapeutic resistance against PARPi in IDHmut brain tumors. This finding reveals a novel mechanistic link between DNA damage repair stress and angiogenic signaling, positioning Prickle4 as a central mediator of adaptive resistance. Importantly, our data suggests that the pro‐angiogenic microenvironment induced by Prickle4 can undermine the long‐term efficacy of PARPi therapy, despite the initial sensitivity of IDHmut tumors. These insights not only highlight Prickle4 as a potential biomarker for predicting resistance but also as a promising therapeutic target to enhance PARPi responses (**Figure** **6**).

## 3 Discussion

It was well established that DDR directly stimulates angiogenesis, as observed in the liver of patients with chronic DNA damage and low DDR capacity, where a higher potential of angiogenesis was evident.^[^ ^23^, ^45^ ^]^ However, whether and how DNA damage directly connects with angiogenic pathways is yet to be elucidated.

In this study, we selected veliparib for our in vivo experiments because, although it is known to have relatively weak PARP‐trapping activity, it is a strong inhibitor of PARylation and possesses favorable pharmacological properties for brain tumor studies. Importantly, other clinically approved PARP inhibitors have shown limitations in brain penetration: olaparib, while able to cross the BBB in glioblastoma patients, exhibits an unfavorable brain/plasma ratio in healthy mice,^[^ ^46^ ^]^ and talazoparib displays restricted BBB penetration.^[^ ^47^ ^]^ By contrast, veliparib demonstrates reliable brain availability, and in our previous work,^[^ ^18^ ^]^ we confirmed its efficacy in glioma models. Moreover, PARP inhibitors can show distinct efficacies in different cellular contexts. For instance, some cell lines respond better to veliparib than olaparib.^[^ ^48^ ^]^ These considerations led us to prioritize veliparib in the current study. Nonetheless, our work primarily tested a PARylation inhibitor, and future studies will be needed to evaluate whether PARP‐trapping inhibitors elicit similar or even stronger resistance mechanisms in IDH‐mutant gliomas.

Our in vitro findings demonstrated that hypoxia significantly amplifies DDR defects induced by PARP inhibition. Given that lenvatinib induced tumor hypoxia in our model, we hypothesized that combining lenvatinib with PARPi would enhance therapeutic efficacy by leveraging this hypoxia‐augmented DDR impairment. However, our transcriptomic analyses revealed that PARPi treatment induces pro‐angiogenic changes in the tumor microenvironment, suggesting that DDR defects are not merely tumor‐intrinsic but also elicit extrinsic compensatory mechanisms such as neovascularization. This finding aligns with the known interplay between DNA damage and angiogenesis, further reinforcing the rationale for targeting angiogenesis along with PARPi. Among the pro‐angiogenic changes, we identified Prickle4 as a critical mediator of resistance to PARPi. Knockdown of Prickle4 sensitized tumors to PARPi, underscoring its role as a potential therapeutic target to overcome resistance. Our study spans both intrinsic (DDR‐related) and extrinsic (microenvironmental) mechanisms of PARPi sensitivity and resistance. This comprehensive approach allowed us to not only validate the rationale for combining lenvatinib and PARPi but also uncover critical resistance pathways, such as Prickle4‐driven angiogenesis, that can inform future therapeutic strategies. However, while our findings provide a strong preclinical rationale for combining PARP and angiogenesis inhibitors in IDH‐mutant gliomas, several challenges remain before this approach can be translated into clinical application. Specifically, the dosing, toxicity, and optimal administration schedules for veliparib and lenvatinib require careful optimization. Future studies focusing on these aspects will be critical to ensuring the safety and efficacy of this therapeutic strategy in patients.

Our study showed that the expression of hypoxia‐related genes is upregulated by IDHmut in vitro culture. Interestingly, we also observed that the angiogenesis score in IDHmut patients is lower than that of IDHwt patients. However, this may reflect the difference in malignancy and the growth pattern in these groups, as IDHwt brain tumors present with a distinct mutational landscape and tend to be relatively fast‐growing. Also, these distinct mutations in IDHwt tumors may alter gene expression by their own mechanisms.

The role of the oncometabolite 2‐HG, whether in its S‐ or D‐form enantiomers, in regulating hypoxia‐associated genes has been explored in some contexts, yet with controversies. For instance, the level of hydroxylation on proline residues of HIF‐1α is reduced by D‐2‐HG.^[^ ^49^ ^]^ Consistently, genetically engineered IDH1‐R132H (*Nes‐KI*) mouse embryos showed higher levels of HIF‐1α protein.^[^ ^50^ ^]^ Our results align with these findings and support the consistent inhibitory role of IDHmut on α‐KG dependent enzymes, including proline hydroxylases. Proline hydroxylases are the enzymes catalyzing prolyl‐hydroxylation on prolyl hydroxylase domain‐containing proteins (PHDs, or EGLNs), including HIF‐1α. Such prolyl‐hydroxylation decreased protein stability, and inhibition of proline hydroxylase by 2‐HG stabilized HIF‐1α. However, other studies presented data suggesting that D‐2‐HG, on the contrary, promotes prolyl‐hydroxylation.^[^ ^33^ ^]^ Thus, whether D‐2‐HG leads to HIF accumulation or degradation may be context‐dependent and remains to be resolved with more relevant evidence.

Sensitivity of IDHmut tumors toward PARPi has been shown corroboratively in multiple contexts. However, recent studies suggested that this sensitivity stems from increased replication stresses rather than alterations in the repair pathways.^[^ ^51^ ^]^ This coincides with our previous finding that IDHmut induces genomic instability that accumulates copy number abnormalities.^[^ ^11^ ^]^ Yet, other studies indicated that pathways related to DDR, such as the lysine demethylase families, display abnormal activities in IDHmut tumors.^[^ ^37^, ^52^ ^]^ Thus, despite clues connecting these observations being elusive, distinct mechanisms may underlie synergy between PARPi and antiangiogenic agents in IDHmut tumors. Our finding of Prickle4‐mediated hyperactive angiogenic activities provides a feasible explanation supporting the use of combination drugs. Nevertheless, gliomas and intrahepatic cholangiocarcinomas are tumors with more oxygen supply. It is unclear whether the combination would show efficacy in other IDHmut tumors. A key therapeutic challenge is that Prickle4, based on its predicted function as a structural protein, may not be readily targetable through conventional small‐molecule inhibitors. This raises the need to explore alternative therapeutic strategies. One promising avenue is the use of proteolysis‐targeting chimeras (PROTACs), which selectively degrade proteins of interest through the ubiquitin‐proteasome route. In principle, a Prickle4‐targeted PROTAC could provide an effective means to deplete Prickle4 protein levels and thereby enhance PARPi efficacy in IDH‐mutant gliomas. In parallel, nucleic acid‐based approaches such as RNA interference or antisense oligonucleotides could be designed to suppress Prickle4 expression, and advances in lipid nanoparticle delivery may make such strategies increasingly feasible in the brain tumor context. While these approaches remain conceptual at present, their development stresses that Prickle4 may represent a tractable target through emerging therapeutic modalities. We anticipate that future work aimed at developing such Prickle4‐directed strategies will further strengthen the translational potential of combining PARPi therapy with Prickle4 inhibition.

We observed upregulated PD‐L1 expression specifically in IDHmut tumors post PARPi. The expression level of PD‐L1 by tumor cells is a predictive marker for immune checkpoint inhibitors (ICI).^[^ ^53^ ^]^ Besides, brain tumors, especially the IDHmut subtype, are recognized as immune “cold tumors”. That PARPi treatment evokes lymphocyte infiltration concurrently with the PD‐L1 increase warrants further preclinical studies of whether PARPi increases the response rate toward ICI. These studies require ideal animal models that possess a competent immune system, as well as a microenvironment ensemble that mimics true situations in tumors.

## 4 Conclusion

Taken together, our study highlights several important insights into the biology and therapeutic vulnerabilities of IDHmut gliomas. We demonstrate that IDH mutations modulate hypoxia‐related pathways, angiogenesis, and DNA damage responses in a context‐dependent manner. We further identify Prickle4 as a novel mediator of hyperactive angiogenesis and propose it as a potential therapeutic target through emerging modalities such as PROTACs or RNA‐based strategies. Finally, we show that PARPi treatment in IDHmut tumors induces PD‐L1 expression and lymphocyte infiltration, raising the prospect of combining PARPi with immune checkpoint blockade. Collectively, these findings provide mechanistic and translational rationales for novel combinatorial strategies, opening avenues toward more effective treatments for IDHmut gliomas.

## 5 Experimental Section

### Cell Culture

HEK‐293T and immortalized human astrocyte (IHA) were maintained in DMEM (Dulbecco's Modified Eagle Medium) (Gibco, C11995500BT) supplemented with 10% fetal bovine serum (Biological Industries, #04‐001‐1A) and 1% Penicillin‐Streptomycin Solution (Thermo Fisher, #15070063). HUCCT1 cell line was a kind gift from Dr. Guoyuan Liu (Fudan University), and was maintained in RPMI‐1640 (Gibco, C1187550BT) supplemented with 10% fetal bovine serum (Biological Industries, 04‐001‐1A) and 1% Penicillin‐Streptomycin Solution (Thermo Fisher, 15070063). DF‐1 was purchased from Cell Resource Center, Peking Union Medical College (PCRC, #1101BIR‐PUMC000417) and maintained in MEM (Minimum Essential Medium) supplemented with 20% fetal bovine serum (ExCell Bio, #FSP500) and 1% Penicillin‐Streptomycin Solution (Thermo Fisher, 15070063). Glioma stem‐like cell line GC02 was generated in‐house following procedures in the “Patient Derived Glioma Stem‐like Cell Lines” section and maintained in NeuroCult NS‐A proliferation medium (STEMCELL Technologies, 05751). Expression of IDHmut in GC02 was achieved by viral transduction of a PSLIK‐IDH1R132H (IDHmut) or PSLIK‐neo (EV, Addgene #25735) vector. For knockdown of Prickle4, a shRNA CAATGCCGCCTGGAGACTATTCTCGAGAATAGTCTCCAGGCGGCATTG was cloned into a FUGW vector (Addgene #14883), with the EGFP expression cassette replaced with an RFP cassette. All cell lines were cultured in a 37 °C incubator with 5% CO_2_. Hypoxic culture was done with a hypoxic chamber wherein 1% O_2_ was supplemented. Cell lines were authenticated by short tandem repeat analyses and routinely monitored for mycoplasma contamination.

### Experimental Animals

The Tg (NES‐tv‐a)/ J12Ech mouse strain was introduced from the National Cancer Institute of the United States through a material transfer agreement (MTA Agreement No. 13370221).^[^ ^54^ ^]^ IDHmut brain tumors were generated as described.^[^ ^26^ ^]^ NOD.Cg‐Prkdc^scid^Il2rg^em1Smoc^ (NSG) mice were purchased from the Shanghai Model Organisms Center, Inc. and maintained in a barriered animal facility at Fudan University. All mouse procedures were reviewed and approved by the Institutional Animal Care and Use Committee at FDU strictly following its guidelines (IACUC approval #20210302‐097).

### In Vivo Experiment

For assessment in RCAS‐TVA brain tumors, before the experiment began, shaved mice's head hair was shaved, and their naked skin was treated with 75% alcohol and iodine. Exposed the skull and recognized the bregma point as the coordinate origin. Skull was drilled at the position of 2 mm left and 3 mm down to the bregma on a dorsal view, with an injection depth of 3 mm. Each mouse was injected with 5 × 10^5^ 3uL^−1^ of cells, and the surgical wound was glued with tissue adhesive. Tumorigenesis was monitored by weekly magnetic resonance imaging (MRI). No animals were excluded from treatment regimens. Randomization was based on their tumor sizes on MR images prior to treatment initiation. The administration experiment was conducted in groups according to MRI results, which included the drugs veliparib (intraperitoneal injection, 25 mg kg^−1^d^−1^, Selleck, S1164) and lenvatinib (intraperitoneal injection, 10 mg kg^−1^d^−1^, Selleck, S1004). For analyses of brain tumor specimens, brain tumors were collected at day 12 post‐treatment initiation and fixed in 10% formaldehyde solution, followed by FFPE preparation.

For GC02 xenograft experiments, 5 × 10^5^ 3ul^−1^ GC02‐IDHmut or GC02‐EV transfected with a pHIV‐Luc‐Zsgreen (Addgene #39196) vector were stereotactically injected following the same procedures above. Administration of veliparib (intraperitoneal injection, 25 mg kg^−1^d^−1^) and lenvatinib (intraperitoneal injection, 10 mg kg^−1^d^−1^) were performed, and the survival curve was recorded, with an experimental end set as moribund symptoms.

### Patient Derived Glioma Stem‐like Cell Lines

The use of human brain tissues was approved by the Institutional Review Board policies at Huashan Hospital affiliated to Fudan University (approval #HIRB‐KY2015‐256). Informed consent was obtained from all study participants. Following surgical resection, glioma tumor samples were immediately placed in Hibernate A medium (ThermoFisher, A1247501) and finely minced into small pieces of ≈0.5–1 mm in diameter, followed by Accutase (ThermoFisher, A1110501) digestion. Isolated single cells were maintained in NeuroCult NS‐A proliferation media (Stem Cell Technologies, 05751) supplemented with EGF (20 ng mL^−1^; Stem Cell Technologies, 78006.2), FGF (10 ng mL^−1^; Stem Cell Technologies, 78134.1), and heparin (2 µg mL^−1^, Stem Cell Technologies, 07980). For xenotransplantation, 5 × 10^5^ 3uL^−1^ GC02 cells were stereotactically injected following the procedures above. The GC02 cell line was transfected with a luciferase‐expressing vector (HIV‐Luc‐ZsGreen),^[^ ^18^ ^]^ allowing follow‐up with bioluminescent imaging.

### RNA‐Seq Analysis

We subjected tumor samples after 12 days of treatment, and sent them to Keymed Biosciences Inc. for downstream RNA sequencing. In brief, following RNA isolation with TRIzol, a total amount of 2 µg RNA per sample was used as input material for library preparations. Sequencing libraries were generated per sample with the NEBNext Ultra RNA Library Prep Kit for Illumina (NEB, E7530L) according to the manufacturer's instructions, and subjected to deep sequencing with an Illumina sequencer NovaSeq 6000. The low‐quality reads were filtered out by FastQC,^[^ ^55^ ^]^ and the high‐quality reads for each sample were aligned to the mm10 reference genome by Hisat2.^[^ ^56^ ^]^ Expression level of coding genes was calculated by StringTie^[^ ^57^ ^]^ with Transcripts Per Kilobase Million (TPM) methods, with annotation collected from UCSC (https://genome.ucsc.edu/). Differentially expression genes (DEGs) between each group were calculated using DESeq2.^[^ ^58^ ^]^ Genes with |Fold change (FC)| ≥ 1.5 and false discovery rate (FDR) < 0.05 were considered as DEGs. Functional enrichment analyses were performed using Gene Ontology (GO) and Gene set enrichment analysis (GSEA), and defined functional modules with FDR < 0.05 was considered as significant. R (Version 4.0.5) was used for gene expression visualization analysis.

### Angiogenesis Scoring and Survival Analyses

Survival analysis was conducted to examine the relationship between the expression levels of 26 angiogenesis‐related genes and patient outcomes. First, data derived from the TCGA database included a total of 510 samples with patient survival information, comprising 94 IDHwt and 416 IDHmut samples. The GSVA algorithm was applied to calculate an “angiogenesis score” for each glioma sample based on the expression of the 26 angiogenesis‐related genes, transforming the expression matrix into a gene set‐level matrix. The differences in GSVA scores between IDHmut and IDHwt tumors were assessed using a two‐sided *t*‐test. For survival analyses, patients were divided into high and low angiogenesis score groups based on the angiogenesis score. A five‐year survival analysis was plotted as Kaplan‐Meier curves, and the survival probabilities were compared by the Log‐rank test with a significance threshold set at *p* < 0.05 to assess significant differences. This analysis was performed in R Studio (version 4.0.5) using the “survival” and “survminer” packages.

### Western Blots

Cell pellets were lysed with JS lysis buffer (50 mm HEPES, 150 mm NaCl, 1% Glycerol, 1% Triton X‐100, 1.5 mm MgCl2, 5 mm EGTA) and protein concentrations were determined by DC protein assay kit II (Biorad, 5000112). Proteins were separated by SDS‐PAGE gels and transferred to a PVDF membrane (Amersham). Blots were incubated in blocking buffer (5% milk 0.1% Tween, 10 mm Tris at pH 7.6, 100 mm NaCl) and then with primary antibody either 1 h at room temperature or overnight at 4 °C. Antibodies used for western blots are as follows: IDH1R132H (Dianova, DIA‐H09, 1:500); IDH1(D2H1)(Cell Signaling Technology, 8137, 1:1000); β‐actin (Proteintech, 10004413, 1:1000); Vinculin (Sigma‐Aldrich, V4505, 1:5000); phospho‐VEGFR2 Tyr 1175 (Cell Signaling Technology, 2478s, 1:1000); total VEGFR2 (Cell Signaling Technology, 2479s, 1:1000), HA‐tag (Cell Signaling Technology, 5017, 1:1000); Anti‐FLAG (Cell Signaling Technology, 14793, 1:1000). Blots were incubated with HRP conjugated secondary antibodies (Biotech, 20000339, 20000374)for 1 h at room temperature, followed by exposure with Clarity Western ECL substrate (Bio‐Rad, 1705061), and imaged on a ChemiDoc (Bio‐Rad, 1708370) imaging system.

### Immunohistochemistry

Tissue samples were fixed in 4% PFA, paraffin‐embedded, and sectioned in 6 µm sections. Tissues were deparaffinized in xylene and rehydrated through a series of graded ethanol and water. For histopathological analysis, sections were stained with hematoxylin (Sigma–Aldrich, 51275) and eosin (Sigma–Aldrich, 199540). For immunohistochemistry, paraffin sections underwent antigen retrieval by boiling in 10 mm Sodium citrate, 0.05% Tween 20, pH 6.0, with a microwave at high power for 6 mins, endogenous peroxidase was blocked, and the slides were then incubated in blocking solution (2.5% BSA, 10% goat serum, in PBS). Incubation with primary antibodies was carried out overnight at 4 °C with the following dilution: anti‐Ki‐67 (Abcam, Ab16667, 1:500); Cleaved Caspase 3 (R&D system, MAB835‐SP, 1:500); IDH1R132H (Dianova, DIA‐H09, 1:250). Slides were incubated with secondary antibodies (Abcam, ab205718, ab205719). Finally, slides were dehydrated, cleared, and mounted with a permanent mounting medium (Absin, 96949‐21‐2).

### Immunofluorescence

Brains were fixed with 4% PFA and incubated with 30% sucrose. Each step was done overnight at 4 °C. Brains were then sectioned into 8 µm sections. For tissue culture, coverslips were pretreated with Poly‐L‐Lysine (Beyotime, C0313‐5 mg) and placed into cell culture dishes for cell adherence. The coverslips were fixed in 4% PFA, and the cell membrane was permeabilized with 0.5% Tween 20 + 0.2% Triton X‐100. For immunofluorescent staining, brain sections or coverslips were blocked in 2% BSA for 1 h at room temperature. Incubation with primary antibodies was carried out overnight at 4 °C as follows: γ‐H_2_AX (Millipore, 05‐636, 1:200), CD31 (Cell Signaling Technology, 77699, 1:500), F4/80 (Abcam, ab6640, 1:200), PRICKLE4 (ThermoFisher, PA5‐72730, 1:200). Section and cell slides were incubated by the secondary antibody (Abcam, ab150113, ab150077, ab150119) at room temperature for 1 h. Finally, the slides were sealed and photographed with a fluorescence microscope.

### PCR and Real‐Time PCR

PCR was used to identify the species of DF‐1. The primers are as follows: chicken&pig&bovine F Primer 1: ACCGCGGTCATACGATTAAC, chicken R Primer 1: CGGTATGTACGTGCCTCAGA, pig R Primer 1: GAATTGGCAAGGGTTGGTAA, bovine R Primer 1: AGTGCGTCGGCTATTGTAGG; chicken F Primer 2: AACCTCCTCCAGCGGATAATAAT, chicken R Primer 2: TTTGTTGGTGGCTGCTTGAA; chicken F Primer 3: TGAGAACTACGAGCACAAAC, chicken R Primer 3: GGGCTATTGAGCTCACTGTT; chicken F Primer 4: ACATAGAACAAACGAAAAAGGATGTG, chicken R Primer 4: CGTCTTAAAGTGAGCTTAGGGCG. The DNA samples were extracted from transgenic mice tissue (TIANGEN, #B0004D‐100). Real‐time PCR experiments were carried out with SYBR green (Vazyme, Q712) to identify mouse genotype, and primers were as follows: Tg‐mouse‐qpcr: 5′‐TTGAGTCAGGTTCCGTGGTGAG‐3′, 5′‐TGCTCTGCCAGCCAGGAATCA‐3′. The primers used in the experiment were synthesized by Beijing Tsingke Biotech, and the purification mode was DSL.

For transcript quantification, real‐time RT‐PCR was performed as described(Andersen et al, 2004) with the following Taqman probes: Ldha (ThermoFisher, Hs01378790_g1), Pdk1 (Hs01561847_m1), Pgk1 (Hs00943178_g1), Slc2a1 (Hs00892681_m1), Vegfa (Hs00900055_m1) and Ca9 (Hs00154208_m1) as internal control; for mouse tumors, Kdm5a (Mm00524457_m1), Prickle4 (Mm04933143_m1), Ddx3y (Mm00465349_m1), Ccl24 (Mm00444700_g1) and Esr1 (Mm00433149_m1) as internal control.

### Comet Assay

Comet assays were performed using OxiSelect Comet Assay Kit (Cell Biolabs, STA‐350) following the manufacturer's instructions. Briefly, a mixture of cell suspension with agarose was dropped onto slides and then lysed with the lysis buffer provided with the kit. Electrophoresis was performed at 13 V for 40 min, followed by staining with Vista Green DNA Dye. Subsequently, the slides were photographed with a fluorescence microscope (ZEISS Axio ImagerA2) and analyzed with the OpenComet plug‐in in ImageJ (Gyori et al, 2021).

### Clonogenic Assay

Cells in the exponential growth phase were counted and seeded at 100, 200, and 400 cells into a six‐well plate. After 2‐3 weeks, plates were fixed with PFA, washed with PBS, and stained with 0.02% crystal violet (Sigma‐Aldrich, C0775) for 1 min, followed by extensive washes with PBS and tap water. Colonies were counted and photographed for statistics.

### Tube Formation Assay

IHA cells were treated with veliparib (20 µm) or vehicle control, and the supernatant was collected at 48 h following treatment. 10^5^ HUVEC cells were seeded in a 24‐well culture plate pre‐coated with Growth Factor Reduced Matrigel Matrix (Corning, 354262). The collected culture supernatant was added to the angiogenesis assay plate and incubated for 6 h, followed by image capture and analysis of in vivo angiogenic activity by quantifying the number of interconnected branching points.

### Statistics

Data were represented as mean ± SEM or SD as specified in the figure legends. For the calculation of the P value, data were analysed by either an unpaired Student's *t*‐test or a 1‐way ANOVA. Survival curves were analyzed by the log‐rank (Mantel‐Cox) test. P values were either directly represented in the figures or represented as: ^*^ *p* < 0.05; ^**^ *p* < 0.01; ^***^ *p* < 0.001; ^****^ *p* < 0.0001, and ns: not significant. The data were analyzed by GraphPad Prism 7.

### Study Approval

All mouse procedures were reviewed and approved by the Institutional Animal Care and Use Committee at FDU strictly following its guidelines (IACUC approval #20210302‐097). The use of human brain tissues was approved by the Institutional Review Board policies at Huashan Hospital affiliated to Fudan University (approval #HIRB‐KY2015‐256).

### Data Availability

RNA sequencing data for mouse brain tumors have been uploaded to Gene Expression Omnibus (GEO) with accession number GSE249818. Supporting data values for figures in the main manuscript and supplement materials were provided in the Supporting Data Values file. All data and materials related to this manuscript will be made available upon request, utilizing material transfer agreements when appropriate.

## Conflict of Interest

T.A.C. is a co‐founder of Gritstone Oncology and holds equity. T.A.C. holds equity in An2H. T.A.C. acknowledges grant funding from Bristol‐Myers Squibb, AstraZeneca, Illumina, Pfizer, An2H, and Eisai. T.A.C. has served as an advisor for Bristol‐Myers, MedImmune, Squibb, Illumina, Eisai, AstraZeneca, and An2H. T.A.C. is an inventor on intellectual property and a patent held by MSKCC on using tumor mutation burden to predict immunotherapy response, which has been licensed to PGDx.

## Author Contributions

J.Y., H.Y., Y.Y., and C.Z. contributed equally to this work. Y.W. designed the experiments. J.Y., H.Y., Y.Y., and S.C. collected the data. Y.L. made the pathological determinations. C.Z. and Z.Z. performed analyses of RNA sequencing. Y.Y., Z.Q., and L.C. provided support of clinical samples. all authors have made substantial contributions to the imaging, data analyses, and figure/manuscript preparation.

## Supporting information



Supporting Information

## Data Availability

The data that support the findings of this study are openly available in Gene Expression Omnibus at https://www.omicsdi.org/dataset/geo/GSE249818, reference number 249818.

## References

[1] J. L. Hopkins , L. Lan , L. Zou , Genes Dev. 2022, 36, 278. 35318271 10.1101/gad.349431.122 PMC8973847

[2] F. J. Groelly , M. Fawkes , R. A. Dagg , A. N. Blackford , M. Tarsounas , Nat. Rev. Cancer 2023, 23, 78. 36471053 10.1038/s41568-022-00535-5

[3] R. Huang , P. K Zhou , Signal Transduct. Target Ther. 2021, 6, 254. 34238917 10.1038/s41392-021-00648-7 PMC8266832

[4] C. Underhill , M. Toulmonde , H. Bonnefoi , Ann. Oncol. 2011, 22, 268. 20643861 10.1093/annonc/mdq322

[5] J. Murai , P. Y. Brcaness , Cancer Res. 2023, 83, 1173. 37057596 10.1158/0008-5472.CAN-23-0628

[6] C. J. Lord , A. Ashworth , Nat. Rev. Cancer 2016, 16, 110. 26775620 10.1038/nrc.2015.21

[7] M. Weller , M. van den Bent , M. Preusser , E. Le Rhun , J. C. Tonn , G. Minniti , M. Bendszus , C. Balana , O. Chinot , L. Dirven , P. French , M. E. Hegi , A. S. Jakola , M. Platten , P. Roth , R. Rudà , S. Short , M. Smits , M. J. B. Taphoorn , A. von Deimling , M. Westphal , R. Soffietti , G. Reifenberger , W. Wick , Nat. Rev. Clin. Oncol. 2021, 18, 170. 33293629 10.1038/s41571-020-00447-z PMC7904519

[8] V. Fougner , B. Hasselbalch , U. Lassen , J. Weischenfeldt , H. S. Poulsen , T. Urup , Neurooncol. Adv. 2022, 4, vdac157. 36325372 10.1093/noajnl/vdac157 PMC9616055

[9] C. Lu , P. S. Ward , G. S. Kapoor , D. Rohle , S. Turcan , O. Abdel‐Wahab , C. R. Edwards , R. Khanin , M. E. Figueroa , A. Melnick , K. E. Wellen , D. M. O'Rourke , S. L. Berger , T. A. Chan , R. L. Levine , I. K. Mellinghoff , C. B. Thompson , Nature 2012, 483, 474. 22343901 10.1038/nature10860 PMC3478770

[10] S. Turcan , D. Rohle , A. Goenka , L. A. Walsh , F. Fang , E. Yilmaz , C. Campos , A. W. M. Fabius , C. Lu , P. S. Ward , C. B. Thompson , A. Kaufman , O. Guryanova , R. Levine , A. Heguy , A. Viale , L. G. T. Morris , J. T. Huse , I. K. Mellinghoff , T. A. Chan , Nature 2012, 483, 479. 22343889 10.1038/nature10866 PMC3351699

[11] S. Turcan , V. Makarov , J. Taranda , Y. Wang , A. W. M. Fabius , W. Wu , Y. Zheng , N. El‐Amine , S. Haddock , G. Nanjangud , H. C. LeKaye , C. Brennan , J. Cross , J. T. Huse , N. L. Kelleher , P. Osten , C. B. Thompson , T. A. Chan , Nat. Genet. 2018, 50, 62. 29180699 10.1038/s41588-017-0001-z PMC5769471

[12] R. J. Molenaar , D. Botman , M. A. Smits , V. V. Hira , S. A. van Lith , J. Stap , P. Henneman , M. Khurshed , K. Lenting , A. N. Mul , D. Dimitrakopoulou , C. M. van Drunen , R. A. Hoebe , T. Radivoyevitch , J. W. Wilmink , J. P. Maciejewski , W. P. Vandertop , W. P. Leenders , F. E. Bleeker , C. J. van Noorden , Cancer Res. 2015, 75, 4790. 26363012 10.1158/0008-5472.CAN-14-3603

[13] T. A. Johannessen , J. Mukherjee , P. Viswanath , S. Ohba , S. M. Ronen , R. Bjerkvig , R. O. Pieper , Mol. Cancer Res. 2016, 14, 976. 27430238 10.1158/1541-7786.MCR-16-0141 PMC5065766

[14] P. L. Sulkowski , C. D. Corso , N. D. Robinson , S. E. Scanlon , K. R. Purshouse , H. Bai , Y. Liu , R. K. Sundaram , D. C. Hegan , N. R. Fons , G. A. Breuer , Y. Song , K. Mishra‐Gorur , H. M. De Feyter , R. A. de Graaf , Y. V. Surovtseva , M. Kachman , S. Halene , M. Günel , P. M. Glazer , R. S. Bindra , Sci. Transl. Med. 2017, 9, aal2463. 10.1126/scitranslmed.aal2463 PMC5435119 28148839

[15] I. K. Mellinghoff , M. J. van den Bent , D. T. Blumenthal , M. Touat , K. B. Peters , J. Clarke , J. Mendez , S. Yust‐Katz , L. Welsh , W. P. Mason , F. Ducray , Y. Umemura , B. Nabors , M. Holdhoff , A. F. Hottinger , Y. Arakawa , J. M. Sepulveda , W. Wick , R. Soffietti , J. R. Perry , P. Giglio , M. de la Fuente , E. A. Maher , S. Schoenfeld , D. Zhao , S. S. Pandya , L. Steelman , I. Hassan , P. Y. Wen , T. F. Cloughesy , N. Engl. J. Med. 2023, 389, 589. 37272516 10.1056/NEJMoa2304194 PMC11445763

[16] A. Natsume , Y. Arakawa , Y. Narita , K. Sugiyama , N. Hata , Y. Muragaki , N. Shinojima , T. Kumabe , R. Saito , K. Motomura , Y. Mineharu , Y. Miyakita , F. Yamasaki , Y. Matsushita , K. Ichimura , K. Ito , M. Tachibana , Y. Kakurai , N. Okamoto , T. Asahi , S. Nishijima , T. Yamaguchi , H. Tsubouchi , H. Nakamura , R. Nishikawa , Neuro. Oncol. 2023, 25, 326. 35722822 10.1093/neuonc/noac155 PMC9925696

[17] N. R. Alshiekh , L. A. F. M. I De , Curr. Neurol. Neurosci. Rep. 2023, 23, 225. 37060388 10.1007/s11910-023-01265-3 PMC10182950

[18] Y. Wang , A. T. Wild , S. Turcan , W. H. Wu , C. Sigel , D. S. Klimstra , X. Ma , Y. Gong , E. C. Holland , J. T. Huse , T. A. Chan , Sci. Adv. 2020, 6, aaz3221. 10.1126/sciadv.aaz3221 PMC7176409 32494639

[19] F. J. Núñez , F. M. Mendez , P. Kadiyala , M. S. Alghamri , M. G. Savelieff , M. B. Garcia‐Fabiani , S. Haase , C. Koschmann , A.‐A. Calinescu , N. Kamran , M. Saxena , R. Patel , S. Carney , M. Z. Guo , M. Edwards , M. Ljungman , T. Qin , M. A. Sartor , R. Tagett , S. Venneti , J. Brosnan‐Cashman , A. Meeker , V. Gorbunova , L. Zhao , D. M. Kremer , L. Zhang , C. A. Lyssiotis , L. Jones , C. J. Herting , J. L. Ross , D. Hambardzumyan , S. Hervey‐Jumper , M. E. Figueroa , P. R. Lowenstein , M. G. Castro , Sci. Transl. Med. 2019, 11, aaq1427. 10.1126/scitranslmed.aaq1427 PMC6400220 30760578

[20] F. Gebauer , M. W. Hentze , Nat. Rev. Mol. Cell Biol. 2004, 5, 827. 15459663 10.1038/nrm1488 PMC7097087

[21] P. L. Majano , J. Clinical investigation. 1998, 101, 1343. 10.1172/JCI774 PMC508711 9525976

[22] J. Guo , J. Yang , Y. Li , Int. J. Clin. Exp. Med. 2015, 8, 8977. 26309550 PMC4537974

[23] W. Wang , S. Dang , Y. Li , M. Sun , X. Jia , R. Wang , J. Liu , PLoS One 2013, 8, 60178. 10.1371/journal.pone.0060178 PMC3618171 23577090

[24] I. Ray‐Coquard , P. Pautier , S. Pignata , D. Pérol , A. González‐Martín , R. Berger , K. Fujiwara , I. Vergote , N. Colombo , J. Mäenp , F. Selle , J. Sehouli , D. Lorusso , E. M. Guerra Alía , A. Reinthaller , S. Nagao , C. Lefeuvre‐Plesse , U. Canzler , G. Scambia , A. Lortholary , F. Marmé , P. Combe , N. de Gregorio , M. Rodrigues , P. Buderath , C. Dubot , A. Burges , B. You , E. Pujade‐Lauraine , P. Harter , N. Engl. J. Med. 2019, 381, 2416. 31851799 10.1056/NEJMoa1911361

[25] M. Ceccarelli , F. P. Barthel , T. M. Malta , T. S. Sabedot , S. R. Salama , B. A. Murray , O. Morozova , Y. Newton , A. Radenbaugh , S. M. Pagnotta , S. Anjum , J. Wang , G. Manyam , P. Zoppoli , S. Ling , A. A. Rao , M. Grifford , A. D. Cherniack , H. Zhang , L. Poisson , C. G. Carlotti Jr , D. P. da Cunha Tirapelli , A. Rao , T. Mikkelsen , C. C. Lau , W. K. A. Yung , R. Rabadan , J. Huse , D. J. Brat , N. L. Lehman , J. S. Barnholtz‐Sloan , S. Zheng , K. Hess , G. Rao , M. Meyerson , R. Beroukhim , L. Cooper , R. Akbani , M. Wrensch , D. Haussler , K. D. Aldape , P. W. Laird , D. H. Gutmann , TCGA Research Network , H. Noushmehr , A. Iavarone , R. G. W. Verhaak , Cell 2016, 164, 550. 26824661 10.1016/j.cell.2015.12.028 PMC4754110

[26] N. M. Amankulor , Y. Kim , S. Arora , J. Kargl , F. Szulzewsky , M. Hanke , D. H. Margineantu , A. Rao , H. Bolouri , J. Delrow , D. Hockenbery , A. M. Houghton , E. C. Holland , Genes Dev. 2017, 31, 774. 28465358 10.1101/gad.294991.116 PMC5435890

[27] X. Qing , W. Xu , S. Liu , Z. Chen , C. Ye , Y. Zhang , Front Immunol. 2022, 13, 843077. 35273618 10.3389/fimmu.2022.843077 PMC8901990

[28] O. L. Chinot , W. Wick , W. Mason , R. Henriksson , F. Saran , R. Nishikawa , A. F. Carpentier , K. Hoang‐Xuan , P. Kavan , D. Cernea , A. A. Brandes , M. Hilton , L. Abrey , T. Cloughesy , N. Engl. J. Med. 2014, 370, 709. 24552318 10.1056/NEJMoa1308345

[29] M. R. Gilbert , J. J. Dignam , T. S. Armstrong , J. S. Wefel , D. T. Blumenthal , M. A. Vogelbaum , H. Colman , A. Chakravarti , S. Pugh , M. Won , R. Jeraj , P. D. Brown , K. A. Jaeckle , D. Schiff , V. W. Stieber , D. G. Brachman , M. Werner‐Wasik , I. W. Tremont‐Lukats , E. P. Sulman , K. D. Aldape , W. J. Curran , M. P. Mehta , N. Engl. J. Med. 2014, 370, 699. 24552317 10.1056/NEJMoa1308573 PMC4201043

[30] U. Herrlinger , N. Schäfer , J. P. Steinbach , A. Weyerbrock , P. Hau , R. Goldbrunner , F. Friedrich , V. Rohde , F. Ringel , U. Schlegel , M. Sabel , M. W. Ronellenfitsch , M. Uhl , J. Maciaczyk , S. Grau , O. Schnell , M. Hänel , D. Krex , P. Vajkoczy , R. Gerlach , R.‐D. Kortmann , M. Mehdorn , J. Tüttenberg , R. Mayer‐Steinacker , R. Fietkau , S. Brehmer , F. Mack , M. Stuplich , S. Kebir , et al., J. Clin. Oncol. 2016, 34, 1611. 26976423 10.1200/JCO.2015.63.4691

[31] A. M. Intlekofer , R. G. Dematteo , S. Venneti , L. W. S. Finley , C. Lu , A. R. Judkins , A. S. Rustenburg , P. B. Grinaway , J. D. Chodera , J. R. Cross , C. B. Thompson , Cell Metab. 2015, 22, 304. 26212717 10.1016/j.cmet.2015.06.023 PMC4527873

[32] S. P. Burr , A. S. H. Costa , G. L. Grice , R. T. Timms , I. T. Lobb , P. Freisinger , R. B. Dodd , G. Dougan , P. J. Lehner , C. Frezza , J. A. Nathan , Cell Metab. 2016, 24, 740. 27923773 10.1016/j.cmet.2016.09.015 PMC5106373

[33] P. Koivunen , S. Lee , C. G. Duncan , G. Lopez , G. Lu , S. Ramkissoon , J. A. Losman , P. Joensuu , U. Bergmann , S. Gross , J. Travins , S. Weiss , R. Looper , K. L. Ligon , R. G. W. Verhaak , H. Yan , W. G. Kaelin Jr , Nature 2012, 483, 484. 22343896 10.1038/nature10898 PMC3656605

[34] K. Tateishi , H. Wakimoto , A. J. Iafrate , S. Tanaka , F. Loebel , N. Lelic , D. Wiederschain , O. Bedel , G. Deng , B. Zhang , T. He , X. Shi , R. E. Gerszten , Y. Zhang , J. J. Yeh , W. T. Curry , D. Zhao , S. Sundaram , F. Nigim , M. V. A. Koerner , Q. Ho , D. E. Fisher , E. M. Roider , L. V. Kemeny , Y. Samuels , K. T. Flaherty , T. T. Batchelor , A. S. Chi , D. P. Cahill , Cancer Cell 2015, 28, 773. 26678339 10.1016/j.ccell.2015.11.006 PMC4684594

[35] P. Metellus , C. Colin , D. Taieb , E. Guedj , I. Nanni‐Metellus , A. M. de Paula , C. Colavolpe , S. Fuentes , H. Dufour , M. Barrie , O. Chinot , L. '. Ouafik , D. Figarella‐Branger , J. Neurooncol. 2011, 105, 591. 21643985 10.1007/s11060-011-0625-2

[36] D. R. Wise , P. S. Ward , J. E. S. Shay , J. R. Cross , J. J. Gruber , U. M. Sachdeva , J. M. Platt , R. G. DeMatteo , M. C. Simon , C. B. Thompson , Proc. Natl. Acad. Sci. U S A 2011, 108, 19611. 22106302 10.1073/pnas.1117773108 PMC3241793

[37] P. L. Sulkowski , S. Oeck , J. Dow , N. G. Economos , L. Mirfakhraie , Y. Liu , K. Noronha , X. Bao , J. Li , B. M. Shuch , M. C. King , R. S. Bindra , P. M. Glazer , Nature 2020, 582, 586. 32494005 10.1038/s41586-020-2363-0 PMC7319896

[38] P. Carmeliet , Nat. Med. 2003, 9, 653. 12778163 10.1038/nm0603-653

[39] D. An , S. Banerjee , J. M Lee , Cancer Treat. Rev. 2021, 98, 102224. 34051628 10.1016/j.ctrv.2021.102224 PMC8217312

[40] J. Bou‐Gharios , G. Noël , H. Burckel , Cell Death Dis. 2024, 15, 503. 39003252 10.1038/s41419-024-06904-2 PMC11246422

[41] L. M. Geraldo , C. Garcia , A. C. C. daFonseca , L. G. F. Dubois , T. C. L. de Sampaio ESpohr , D. Matias , E. S. de Camargo Magalhães , R. F. do Amaral , B. G. da Rosa , I. Grimaldi , F. Leser , J. M. Janeiro , L. Macharia , C. Wanjiru , C. M. Pereira , V. Moura‐Neto , C. Freitas , F. R. S. Lima , Trends in Cancer 2019, 5, 46.30616755

[42] S. Goel , A. H. Wong , R. K Jain , Perspect. Med. 2012, 2, a006486. 10.1101/cshperspect.a006486 PMC3282493 22393532

[43] R. K Jain , Nat. Med. 2001, 7, 987. 11533692 10.1038/nm0901-987

[44] A. L. Magnussen , I. G. Mills , Br. J. Cancer 2021, 125, 324. 33828258 10.1038/s41416-021-01330-z PMC8329166

[45] V. Pastukh , J. T. Roberts , D. W. Clark , G. C. Bardwell , M. Patel , A.‐B. Al‐Mehdi , G. M. Borchert , M. N. Gillespie , Am J. Physiol. Lung Cell Mol. Physiol. 2015, 309, L1367. 26432868 10.1152/ajplung.00236.2015 PMC4669343

[46] C. Hanna , K. M. Kurian , K. Williams , C. Watts , A. Jackson , R. Carruthers , K. Strathdee , G. Cruickshank , L. Dunn , S. Erridge , L. Godfrey , S. Jefferies , C. McBain , R. Sleigh , A. McCormick , M. Pittman , S. Halford , A. J. Chalmers , Neuro. Oncol. 2020, 22, 1840. 32347934 10.1093/neuonc/noaa104 PMC7746945

[47] S. H. Kizilbash , S. K. Gupta , K. Chang , R. Kawashima , K. E. Parrish , B. L. Carlson , K. K. Bakken , A. C. Mladek , M. A. Schroeder , P. A. Decker , G. J. Kitange , Y. Shen , Y. Feng , A. A. Protter , W. F. Elmquist , J. N. Sarkaria , Mol. Cancer Ther. 2017, 16, 2735. 28947502 10.1158/1535-7163.MCT-17-0365 PMC5716902

[48] B. D. Lehmann , J. A. Bauer , X. Chen , M. E. Sanders , A. B. Chakravarthy , Y. Shyr , J. A. Pietenpol , J. Clin. Invest. 2011, 121, 2750. 21633166 10.1172/JCI45014 PMC3127435

[49] W. Xu , H. Yang , Y. Liu , Y. Yang , P. Wang , S.‐H. Kim , S. Ito , C. Yang , P. Wang , M.‐T. Xiao , L.‐X. Liu , W.‐Q. Jiang , J. Liu , J.‐Y. Zhang , B. Wang , S. Frye , Y. Zhang , Y. H. Xu , Q.‐Y. Lei , K.‐L. Guan , S.‐M. Zhao , Y. Xiong , Cancer Cell 2011, 19, 17. 21251613 10.1016/j.ccr.2010.12.014 PMC3229304

[50] M. Sasaki , C. B. Knobbe , M. Itsumi , A. J. Elia , I. S. Harris , I. I. Chio , R. A. Cairns , S. McCracken , A. Wakeham , J. Haight , A. Y. Ten , B. Snow , T. Ueda , S. Inoue , K. Yamamoto , M. Ko , A. Rao , K. E. Yen , S. M. Su , T. W. Mak , Genes Dev 2012, 26, 2038. 22925884 10.1101/gad.198200.112 PMC3444730

[51] J.‐M. Schvartzman , G. Forsyth , H. Walch , W. Chatila , A. Taglialatela , B. J. Lee , X. Zhu , S. Gershik , F. V. Cimino , A. Santella , K. Menghrajani , A. Ciccia , R. Koche , F. Sánchez‐Vega , S. Zha , C. B. Thompson , Mol. Cell 2023, 83, 2347. 37311462 10.1016/j.molcel.2023.05.026 PMC10845120

[52] K. Gunn , M. Myllykoski , J. Z. Cao , M. Ahmed , B. Huang , B. Rouaisnel , B. H. Diplas , M. M. Levitt , R. Looper , J. G. Doench , K. L. Ligon , H. I. Kornblum , S. K. McBrayer , H. Yan , C. Duy , L. A. Godley , P. Koivunen , J.‐A. Losman , Cancer Discov. 2023, 13, 1478. 36847506 10.1158/2159-8290.CD-22-0825 PMC10238656

[53] J. Zouein , C. Kesrouani , H. R. Kourie , Immunotherapy 2021, 13, 1053. 34190579 10.2217/imt-2020-0336

[54] E. C. Holland , W. P. Hively , V. Gallo , H. E. Varmus , Genes Dev. 1998, 12, 3644. 9851971 10.1101/gad.12.23.3644 PMC317261

[55] S. W. Wingett , S. Andrews , F1000Research 2018, 7, 1338. 30254741 10.12688/f1000research.15931.1 PMC6124377

[56] D. Kim , J. M. Paggi , C. Park , C. Bennett , S. L Salzberg , Nat. Biotechnol. 2019, 37, 907. 31375807 10.1038/s41587-019-0201-4 PMC7605509

[57] M. Pertea , G. M. Pertea , C. M. Antonescu , T. C. Chang , J. T. Mendell , S. L. Salzberg , Nat. Biotechnol. 2015, 33, 290. 25690850 10.1038/nbt.3122 PMC4643835

[58] M. I. Love , W. Huber , S. Anders , Genome Biol. 2014, 15, 550. 25516281 10.1186/s13059-014-0550-8 PMC4302049

