# Supplementary material for: Prickle4 Drives Microenvironmental Remodeling and Resistance to Parp Inhibition in IDH‐Mutant Glioma
Source: Adv Sci (Weinh). 2025 Nov 14;13(3):e03866. doi: 10.1002/advs.202503866 (PMC12806547; doi:10.1002/advs.202503866)
Supplement: Supplementary file 1 — Supporting Information [file ADVS-13-e03866-s001.pdf]

Supporting Information

Title: PRICKLE4 DRIVES MICROENVIRONMENTAL REMODELING AND RESISTANCE TO PARP INHIBITION IN IDH-MUTANT GLIOMA

Ju Yang et al.

This PDF file includes:

Figure S1 to S11

Supplementary Figure 1

A

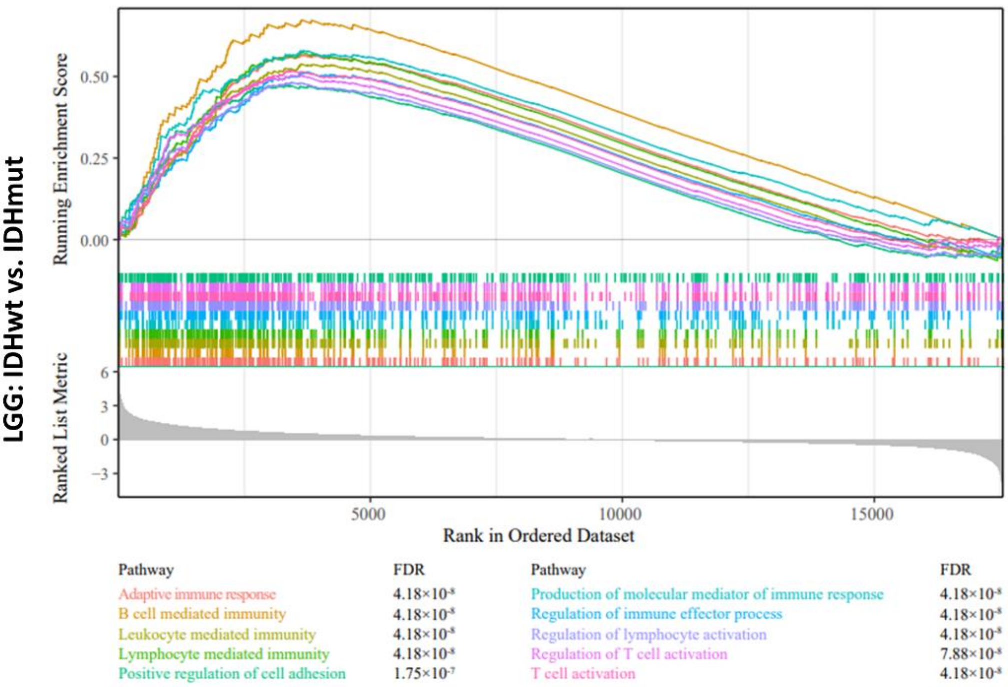

B

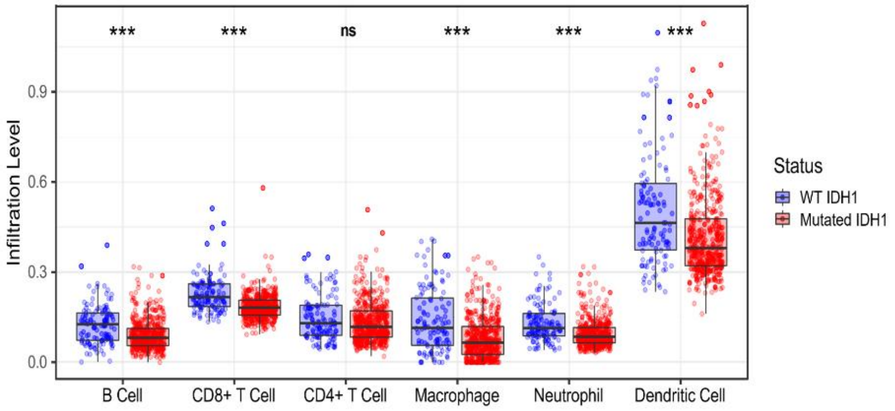

Figure S1. IDH mutations differentially shape the tumor microenvironment. A: GSEA analyses of significantly enriched gene sets related to adaptive immunity. **B**: Infiltration of immune populations in IDHwt and IDHmut tumors estimated from expression data by TIMER. ***: $p < 0.001$, ns: not significant.

Supplementary Figure 2

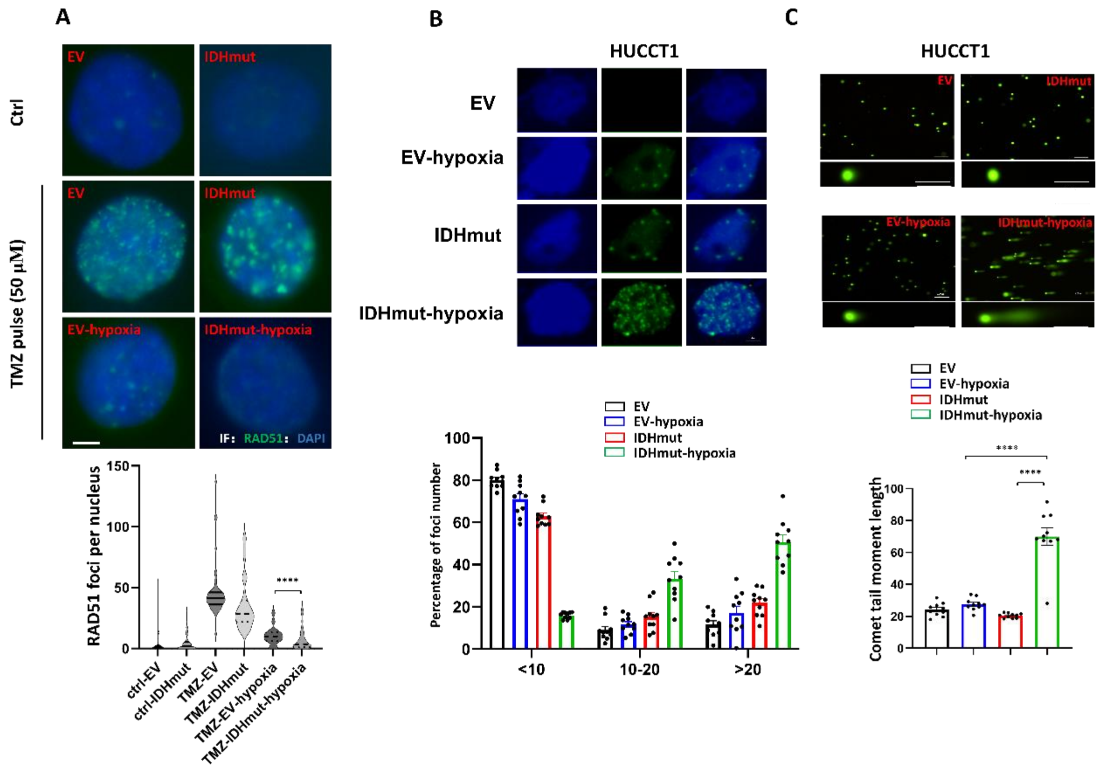

Figure S2. Mutant IDH1 and Hypoxia induce DNA damage synergistically in HUCCT1. A: Immunostaining of RAD51 induction by temozolomide (TMZ) in IDHwt and IDHmut cells under hypoxia (1% O_2) and non-hypoxic (21% O_2) conditions. Scale bar: 2 µm. **B**: Immunostaining of γ-H2AX in HUCCT1-EV and HUCCT1-IDH1mut under hypoxic and non-hypoxic conditions. Scale bar: 10 µm. Bottom: Quantification of (A), representation of percentage of cells with various numbers of foci in the nucleus. N = 10. **C**: Neutral Comet assays determining DNA breaks in HUCCT1-EV and HUCCT1-IDH1mut under hypoxic and non-hypoxic conditions. Scale bars: 200 µm. Bottom: The length of comet tails was measured and represented on the plot. N = 10. *: $p < 0.05$, **: $p < 0.01$, ***: $p < 0.001$, ****: $p < 0.0001$, ns: not significant.

Supplementary Figure 3

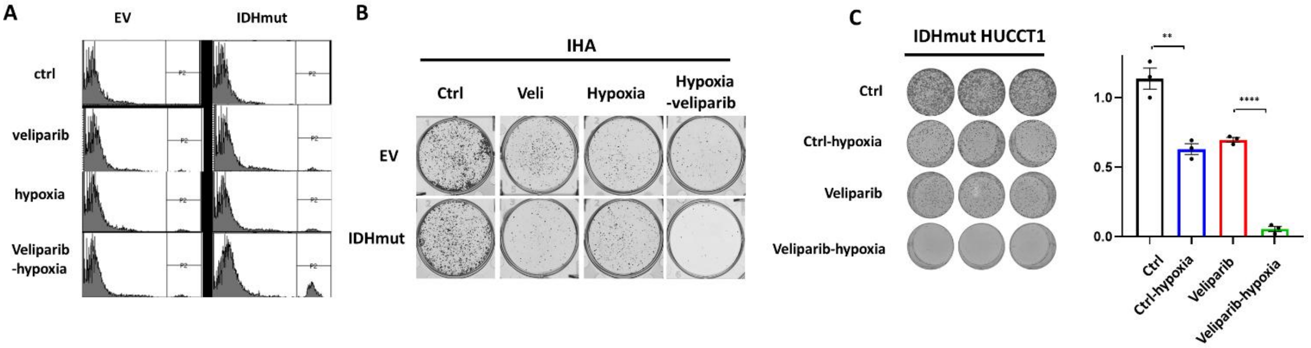

Figure S3. Veliparib treatment and hypoxia inhibit cell proliferation synergistically in IHA and HUCCT1 IDHmut cells. **A**: Representative results of propidium iodide labeling and analyses by flow cytometry. Data were represented in **Figure 1K**. **B**: Representative results of colony formation assay with IHA, treated with or without veliparib, under hypoxic and non-hypoxic conditions. Data were represented in **Figure 1L. C**: Left: Representative results of colony formation assay with HUCCT1-IDH1mut treated with or without veliparib, under hypoxic and non-hypoxic conditions. Right: The colonies of all conditions were quantified and represented on the bar graph. N = 3. *: $p < 0.05$, **: $p < 0.01$, ***: $p < 0.001$, ****: $p < 0.0001$, ns: not significant.

Supplementary Figure 4

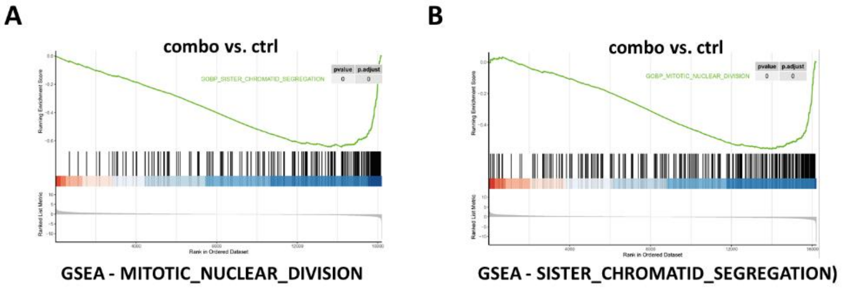

Figure S4. Veliparib and lenvatinib combination induces reduced mitotic activity in IDHmut tumors. GSEA comparing combo vs. ctrl indicated negative enrichment of “mitotic nuclear division” (**A**) and “sister chromatid segregation” **(B)**.

Supplementary Figure 5

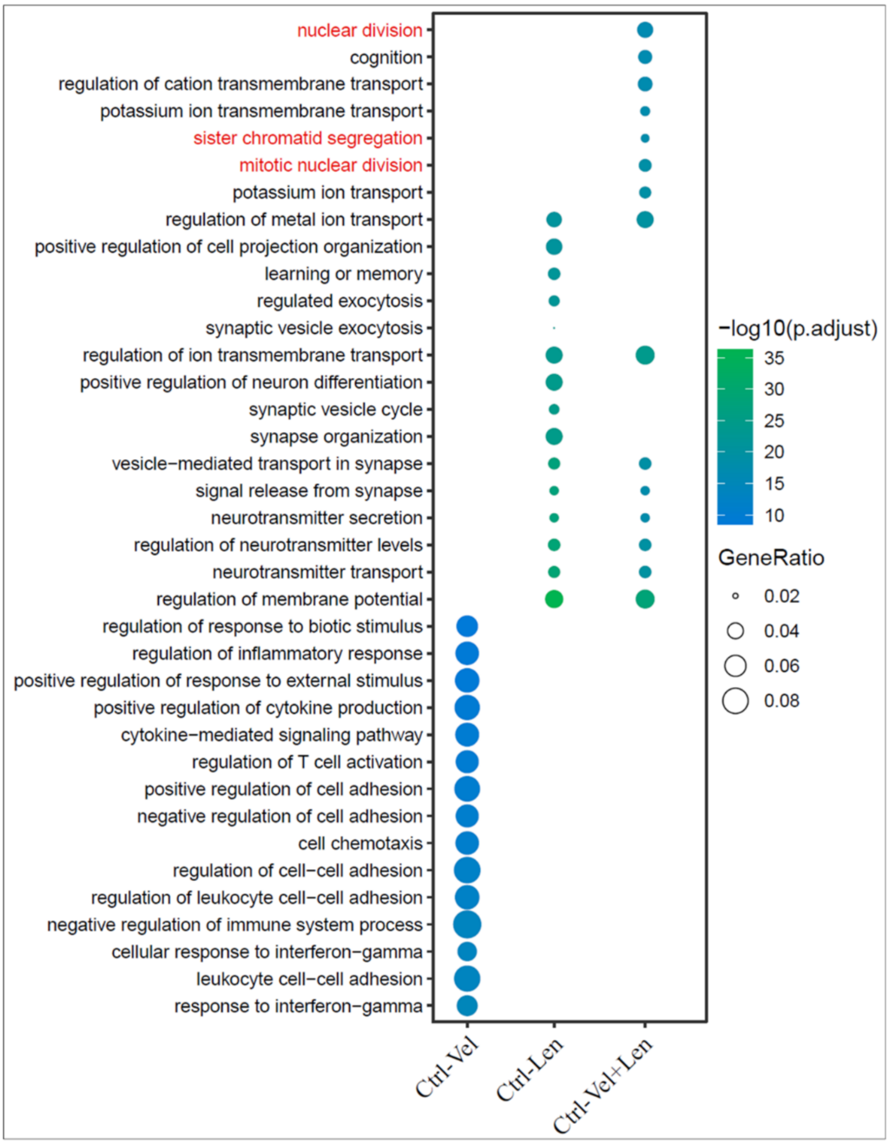

Figure S5. Gene Ontology analyses of IDHmut brain tumors post veliparib and lenvatinib treatments. Gene ratios and adjusted p values were scaled. Comparisons performed were (left to right): veliparib vs. vehicle; lenvatinib vs vehicle and veliparib+ lenvatinib vs. vehicle. Gene sets for “nuclear division”, “sister chromatid segregation” and “mitotic nuclear division” were marked for visualization of inhibited mitosis in veliparib+ lenvatinib vs. vehicle comparison.

Supplementary Figure 6

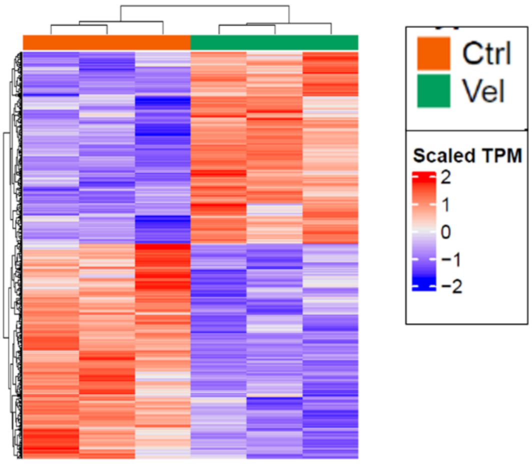

239 up, 271 down

Figure S6: Veliparib induces widespread alterations in gene expression. Gene expression signatures of veliparib (Vel) and vehicle (Ctrl) treated IDHmut brain tumors were determined for differentially expressed genes (DEGs), with cutoff threshold of log2 fold change (FC) > 1.5 and False discovery rate (FDR) < 0.05, and plotted as heatmap. TPM: transcript per million.

Supplementary Figure 7

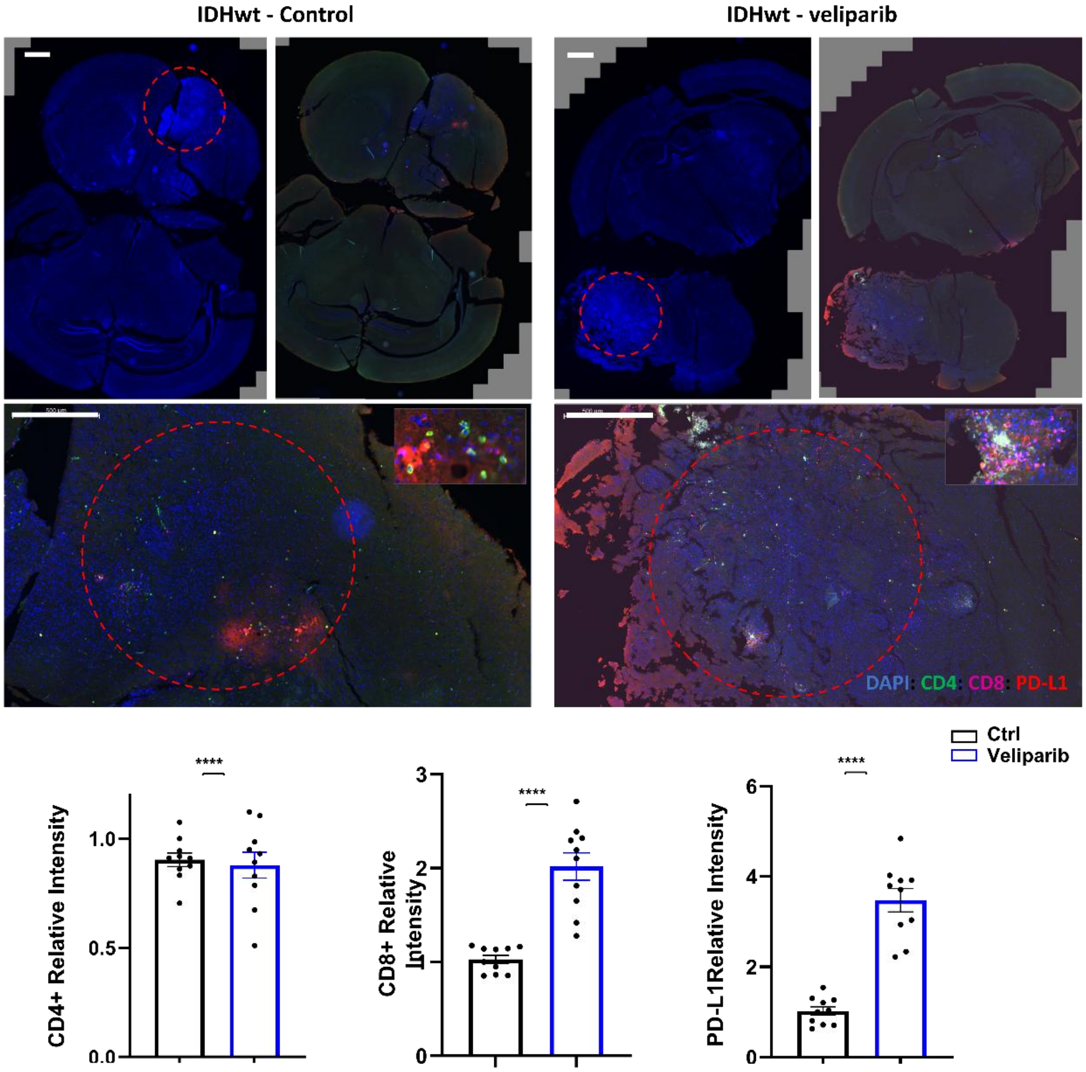

Figure S7: The internal tumor microenvironment changes of mice bearing IDH1wt glioma after treated with veliparib. Upper: Mice bearing IDHwt glioma were sacrificed at day 12 after treatment with veliparib. Immunofluorescent staining of DAPI, CD4, CD8 and PD-L1 were performed. Red circles indicate tumor areas. Scale bar: 500 μm. Lower: Relative intensity of CD4, CD8 and PD-L1 in vehicle and veliparib were quantified and plotted as means ± SEM. N = 10. *: $p < 0.05$, **: $p < 0.01$, ***: $p < 0.001$, ****: $p < 0.0001$, ns: not significant.

Supplementary Figure 8

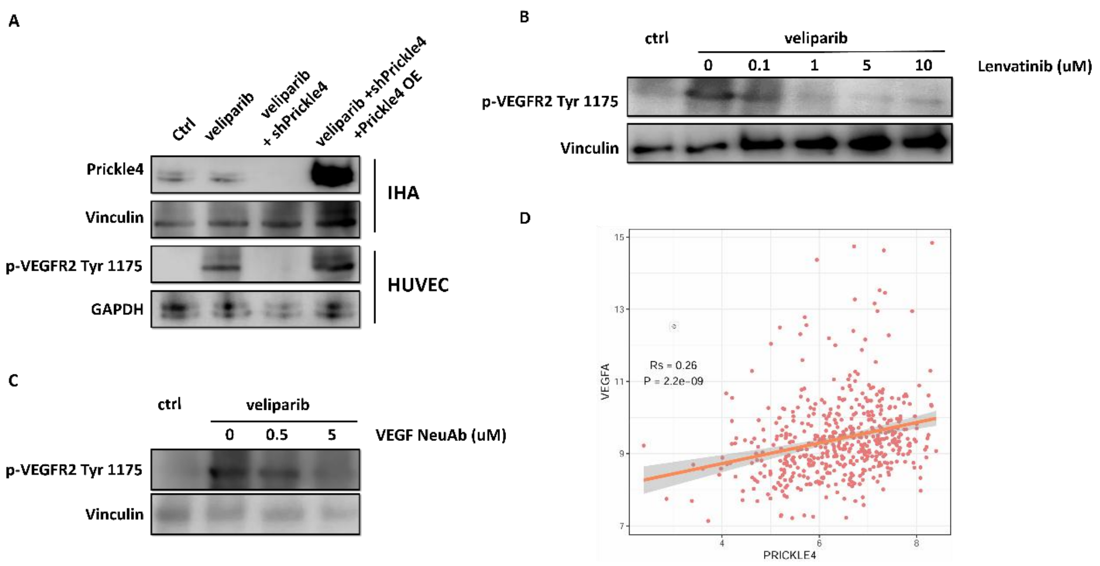

Figure S8: Prickle4 affects VEGFR2 phosphorylation in co-cultured HUVEC cells. **A**: Rescue of Prickle4 expression in knockdown cells restored VEGFR2 phosphorylation in co-cultured HUVEC cells. **B**: HUVEC cells treated with lenvatinib in the co-culture setting showed decreased VEGFR2 phosphorylation at 1, 5 and 10 μM. **C**: Neutralization of VEGF in the conditioned medium by bevacizumab effectively blocked VEGFR2 phosphorylation. **D**: Expression data in the clinical samples showed a significant positive correlation between Prickle4 and VEGFA expression.

Supplementary Figure 9

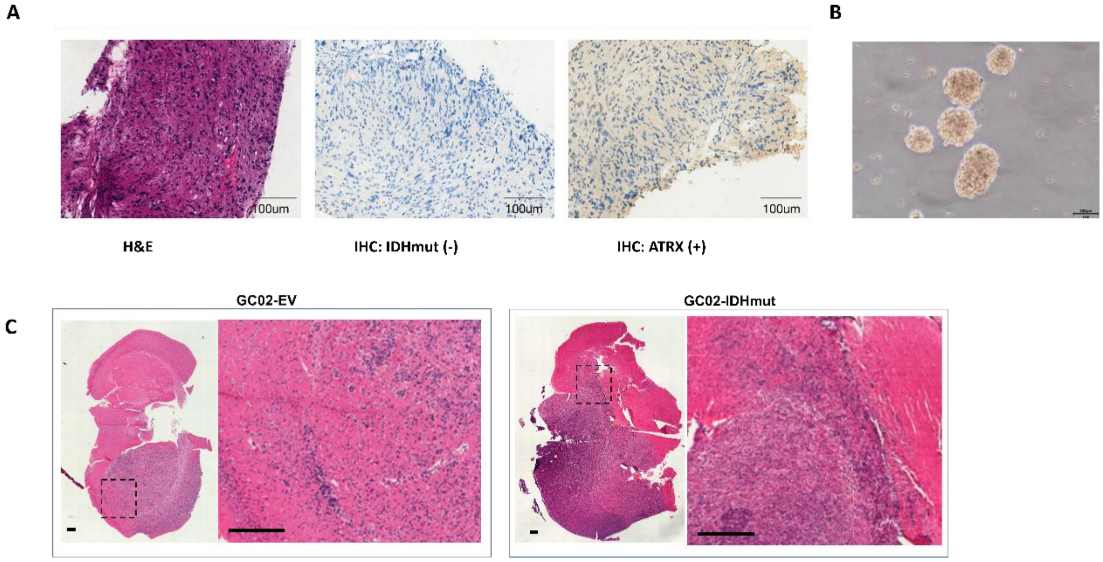

Figure S9: Establishment of GC02 and GC02-IDHmut GSC lines and their xenograft models. A: Tumor resection specimen were subjected for FFPE processing, followed by H&E staining (left) and immunostaining for IDH1mut (middle) and ATRX (right). Scale bars represent 100 µm. **B**: Representative microscopic photograph of suspension GC02 culture. **C**: Scans of whole sections of GC02-EV (left) and GC02-IDHmut (right), showing infiltrative growth pattern. Scale bar: 200 µm.

Supplementary Figure 10

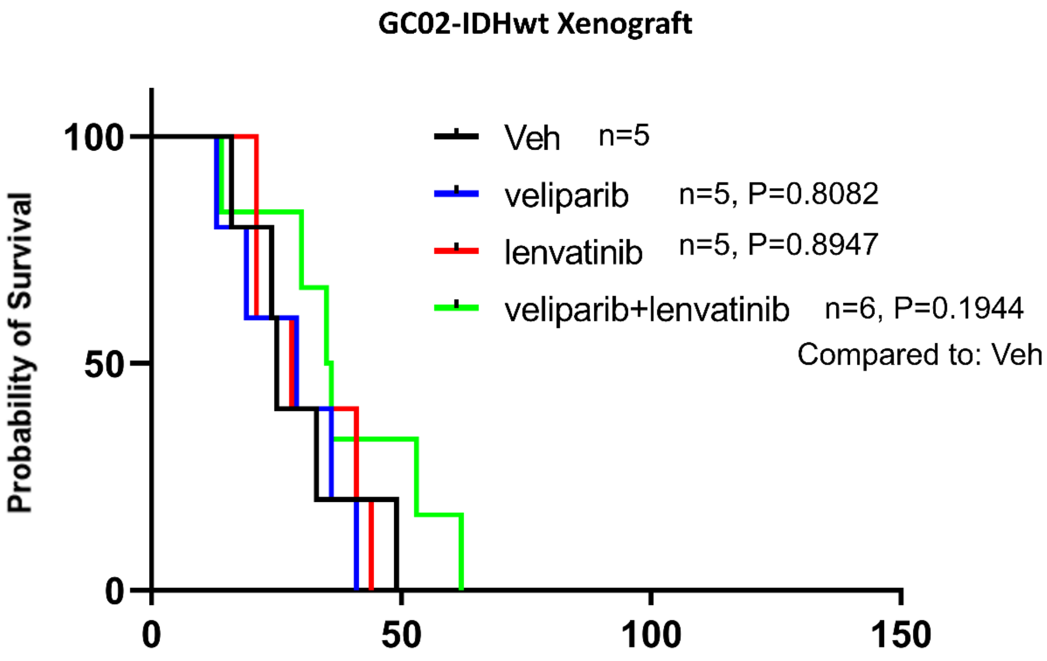

Figure S10. Combination treatment did not extend survival of mice bearing GC02-IDHwt tumors. Kaplan-Meier analysis of mice bearing GC02 xenograft on vehicle, veliparib, Lenvatinib and the combination treatment. P values were determined by log-rank (Mantel-Cox) test. The number of animals per group (n) is represented on the plot.

Supplementary Figure 11

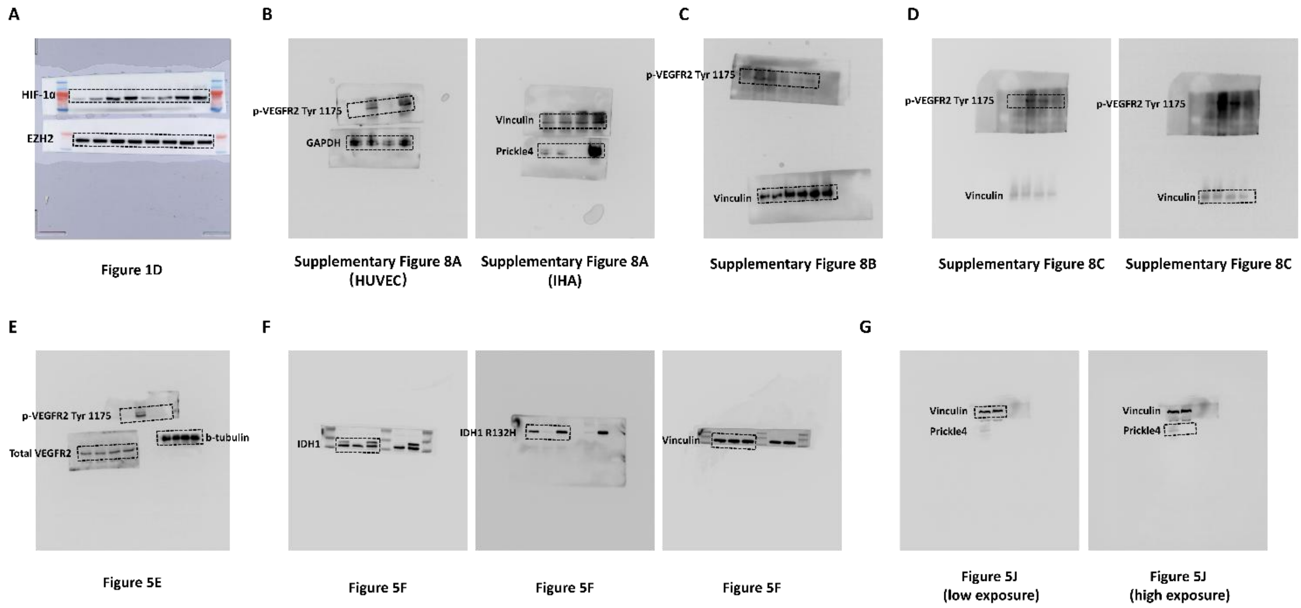

Figure S11. Original uncropped Western blot images related to the study " Prickle4 Drives Microenvironmental Remodeling and Resistance to PARP Inhibition in IDH-Mutant Glioma". A: Blots corresponding to Figure 1D. B: Blots corresponding to Supplementary Figure 8A. C: Blots corresponding to Supplementary Figure 8B. D: Blots corresponding to Supplementary Figure 8C. E: Blots corresponding to Figure 5E. F: Blots corresponding to Figure 5F. G: Blots corresponding to Figure 5J.
